# Pollen longevity and viability dataset: an integrated resource for plant research and conservation

**DOI:** 10.1111/nph.71325

**Published:** 2026-06-02

**Authors:** Louise Winther, Conny Bruun Asmussen Lange, Sergey Rosbakh

**Affiliations:** ^1^ Section of Organismal Biology, Department of Plant and Environmental Sciences University of Copenhagen Frederiksberg 1871 Denmark

**Keywords:** dataset, germinability, germplasm, longevity, pollen, stainability, storage, viability

## Abstract

Pollen viability and longevity are key traits in plant reproduction, with implications for gene flow, crop breeding, and *ex situ* plant conservation. However, published data on these traits remain dispersed across disciplines, languages, and methods, limiting their accessibility and reuse. Here, we present a curated, taxonomically broad dataset compiling pollen viability over time for 274 vascular plant species extracted from 320 primary publications published between 1962 and 2023. The dataset includes germination‐ and staining‐based viability estimates under defined storage conditions, along with metadata on species identity, experimental design, and pollen traits. To facilitate data exploration and application, the full dataset is openly available via an online repository and accompanied by an interactive Shiny App that allows users to filter, visualize, and download customized subsets. This resource provides a structured foundation for comparative analyses of pollen longevity and viability within well‐represented clades and experimental contexts. It supports trait‐based and applied research when combined with external data sources, while also serving as a starting point for future syntheses.

## Graphical abstract

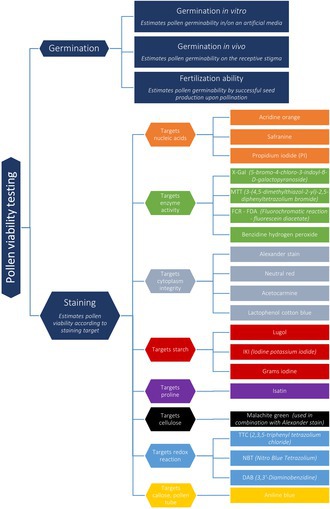

**Table:** 

	Content	
	Summary	1631
I.	Introduction	1631
II.	The pollen longevity and viability dataset	1632
III.	Materials and Methods	1632
IV.	Dataset structure	1634
V.	Current state of the dataset and implications for inference	1634
VI.	Opportunities for synthesis and application of pollen longevity data	1638
	Acknowledgements	1638
	References	1639

## I. Introduction

### 1. Why is information on pollen longevity important

Viable pollen is essential for plant sexual reproduction, acting as the mobile vector that facilitates gene flow among populations (Barnabás & Kovács, 1997; Gowthami *et al*., 2024). For successful fertilization, pollen must remain viable until landing on the stigma and reaching the ovule through pollen tube growth, enduring environmental fluctuations for various periods of time (Mesihovic *et al*., 2016). Pollen longevity, that is, the time during which the pollen remains viable and can produce a functioning pollen tube on the receptive stigma, varies considerably among species (Dafni & Firmage, 2000; Edlund *et al*., 2004). It ranges from minutes in some grasses to several months in roses and palms (Barnabás & Kovács, 1997; Dafni & Firmage, 2000). Pollen longevity is driven by multiple intrinsic and extrinsic factors like pollen morphology, temperature, or relative humidity (Dafni & Firmage, 2000; Althiab‐Almasaud *et al*., 2023).

Because pollen longevity itself has important implications for ecological processes, information on its variation across populations and species under various environmental conditions (e.g. plant growth and pollen storage conditions) is of central importance for plant ecology and biology, agriculture, and conservation efforts (Dafni & Firmage, 2000). As the male gametophyte carrying genetic material, pollen contributes to species diversification and adaptation to changing environments, ensuring fruit and seed production, which also constitute the majority of human nutrition (Althiab‐Almasaud *et al*., 2023; Gowthami *et al*., 2024). Viable pollen is essential for germplasm exchange in agriculture, for example, for crop improvement such as hybridizing individuals that flower at different times (Pacini & Dolferus, 2019). Finally, information on pollen longevity is crucial for conservation purposes both locally and globally, particularly for *ex situ* storage. Pollen storage provides an alternative to conventional seed banking and is especially valuable for species with recalcitrant seeds or for threatened and isolated populations that do not produce viable seeds (Dafni & Firmage, 2000; Gowthami *et al*., 2024; Wolkis *et al*., 2024).

### 2. Approaches to pollen viability testing in pollen longevity research

**Fig. 1:**
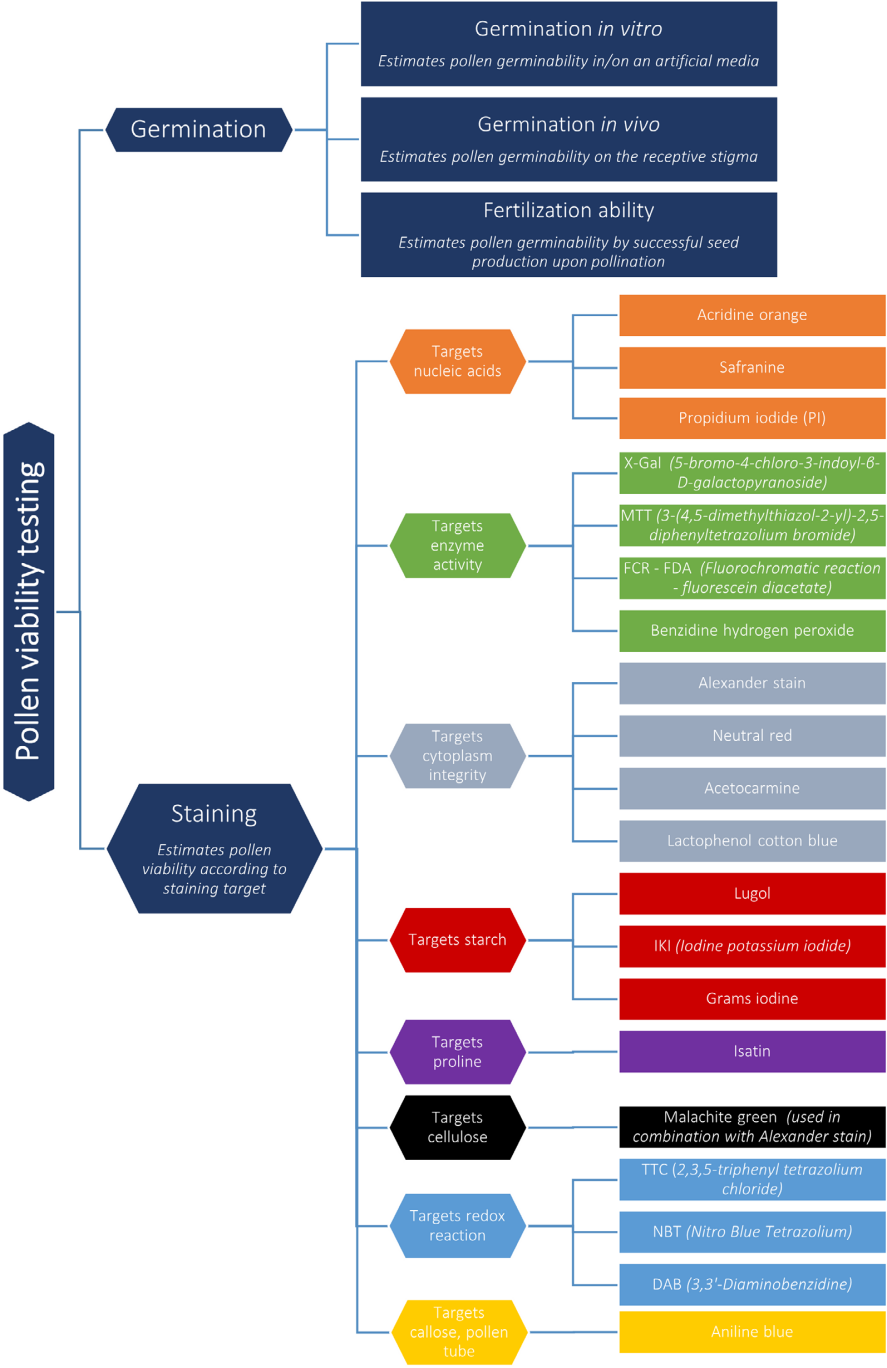
The most common methods to estimate pollen viability. The figure summarizes two approaches for estimating pollen viability: germination tests and staining methods with different target categories. The staining methods are colour‐coded according to the staining target category.

Traditionally, pollen longevity has been assessed by observing pollen ageing under either natural or predefined environmental conditions, continuously estimating the viability over time. The two most common approaches for estimating pollen viability are by germinating or staining pollen (Fig. 1). Pollen germination methods confirm viability by the production of a pollen tube, either *in vitro* in a specialized liquid or on a solid medium, or *in vivo* through fertilization ability (Dafni & Firmage, 2000; Tushabe & Rosbakh, 2021; Althiab‐Almasaud *et al*., 2023). Staining techniques target different anatomical or biochemical features of pollen grains that might indicate their viability; for example, the fluorescein diacetate stain (FDA), which turns fluorescent upon hydrolysis by active esterases in a viable pollen grain. Importantly, all available methods only provide an estimate of pollen viability. Pollen germination methods might underestimate viability as the pollen could still be vigorous and capable of germinating under conditions other than those in the given method (Dafni & Firmage, 2000). Stains have a tendency to overestimate pollen viability as, for example, enzyme activity might still be active after the pollen loses its ability to germinate (Dafni & Firmage, 2000). Moreover, most commonly used pollen viability assays are destructive, meaning that pollen grains used for testing cannot subsequently be used for fertilization or storage, which may reduce the size of small pollen collections when repeated testing is required.

### 3. Knowledge gaps in pollen longevity

The importance of pollen longevity as a key trait in the plant life history cycle was already recognized in the late 19th century, resulting in thousands of publications, particularly on cultivated plants (Mangin, 1886). However, pollen longevity data remains scattered across various journals from different fields, frequently published in different languages, making it challenging for researchers and practitioners to access and utilize this information. Furthermore, existing reviews of pollen longevity do not include the changes in pollen viability over time including corresponding experimental data, but only report simplified estimates, often for a limited number of species (Pfundt, 1909; Knowlton, 1922; Holman & Brubaker, 1926; Barnabás & Kovács, 1997; Dafni & Firmage, 2000). Consequently, as the significance of pollen longevity grows across research fields and conservation efforts, the lack of a comprehensive, accessible resource remains a critical barrier that slows progress in data synthesis, comparative studies, and practical applications.

## II. The pollen longevity and viability dataset

This Community resource provides the first taxonomically broad and methodologically annotated dataset on pollen viability and longevity including more than 21 000 entries. We compiled detailed data for 466 vascular plant species from 243 genera and 87 families world‐wide, covering both wild and cultivated taxa. The information was extracted from 320 primary publications published between 1962 and 2023 in English, German, Korean, Chinese, Portuguese, and Spanish. The dataset includes pollen viability estimates recorded at multiple time points under defined storage and experimental conditions, using both germination‐ and staining‐based approaches. By standardizing and structuring these data, the resource enables comparative research on how pollen longevity varies across taxonomic groups, environmental treatments, and experimental approaches. It supports a wide range of applications: ecologists can investigate how longevity correlates with life history traits or pollination mode; conservation biologists can identify species vulnerable to reproductive failure or target candidates for *ex situ* preservation; and plant breeders can use the data to optimize pollen storage and hybridization protocols (Dafni & Firmage, 2000; Volk, 2011; Pacini & Dolferus, 2019; Althiab‐Almasaud *et al*., 2023; Wolkis *et al*., 2024). To promote accessibility and broad use, the full dataset is openly available through an online repository (https://doi.org/10.5281/zenodo.17661539), accompanied by a Shiny App (https://w1nther.shinyapps.io/Pollen‐longevity/) that allows users to explore, filter, visualize, and download customized subsets of the data. This resource is intended to support future comparative analyses, hypothesis testing, and applied decision‐making across the plant sciences.

## III. Materials and Methods

### 1. Literature search and data extraction

A systematic literature search identifying studies on pollen longevity was conducted following PRISMA guidelines (Supporting Information Fig. S1) (Page *et al*., 2021). We primarily used the Web of Science (www.webofscience.com) database, which provides a broad coverage of curated peer‐reviewed scientific literature and allows reproducible search queries across papers in multiple languages by indexing English metadata (Gusenbauer & Haddaway, 2020; Clarivate™ Web of Science™, 2024). The final search was performed on 13 August 2023, using a topic search (TS) covering titles, abstracts, author keywords and keywords Plus (Clarivate™ Web of Science™, 2024) with the string ‘TS = (“Pollen longevity” OR (Pollen AND (Preservation OR CryoPreservation OR storage)))’. We excluded document types ‘Patent’ and ‘Review article’, the database type ‘Zoological record’, and studies published in research areas ‘Paleontology’, ‘Geology’ and ‘Entomology’ as well as the medical subject heading ‘Immunoglobulin E’. If a primary article referred to data from another article that was not among the included references and fit the inclusion criteria, we searched for it and retrieved it via Google Scholar. All non‐English publications were translated using Google Translate.

The search obtained 3170 articles from 1915 to 2023 in 27 different indexed languages that were checked against the following inclusion criteria:

**(1)** the article must be a primary article (i.e. standard research or conference paper, or a technical report),

**(2)** the article must contain pollen viability estimates provided either in a table or a figure for at least two time points for a set of defined experimental conditions,

**(3)** datapoints of pollen viability and time should be specific, that is, no means across different treatments and/or intervals, and

**(4)** pollen viability should not be estimated in terms of pollen fertilization ability; that is, the ability of pollinated plants to produce a seed set.

**Fig. 2:**
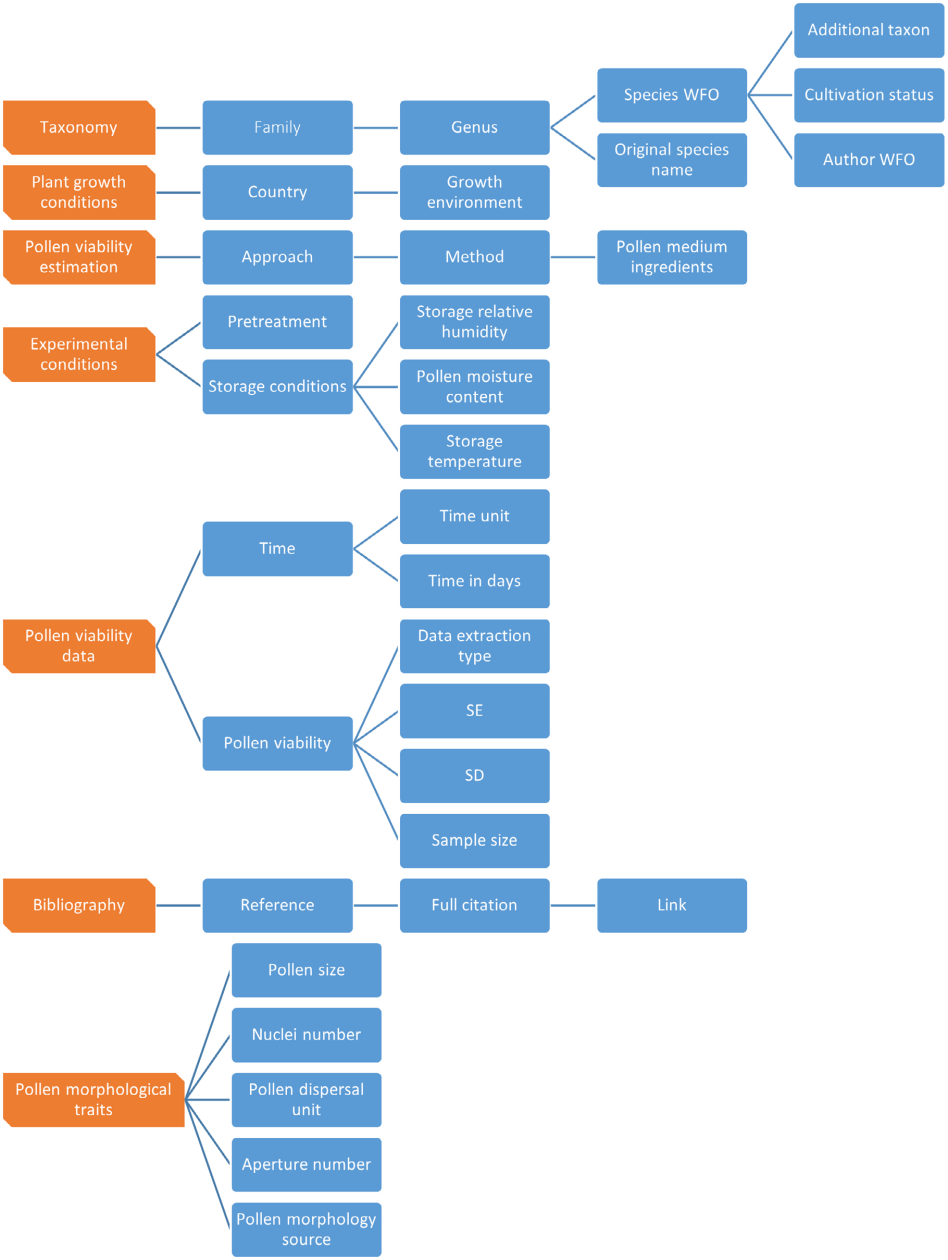
Overview of the variables included in the pollen longevity and viability dataset. Variables (blue boxes) are grouped hierarchically according to their data category (orange boxes). This structure facilitates filtering, comparative analyses, and integration with other trait‐based datasets.

In total, 320 articles (Notes S1) met the inclusion criteria and were further subjected to data extraction of predefined variables as shown in Table 1 and Fig. 2. We prioritized extracting the data from the source available in the following order: by extracting the data from an associated data file, in a supplementary raw file, a table or extracting the data from a figure. Data from figures were extracted using PlotDigitizer (PlotDigitizer, 2025). Using the PlotDigitizer app, viability datapoints (*y*‐axis) were extracted or both viability and time datapoints (*x*‐ and *y*‐axis), if timepoints were not mentioned elsewhere in the article.

**Table 1:** Pollen longevity and viability testing dataset categories with variables and descriptions of such.

Data category	Variable	Description
Taxonomy	Original species name	Species names as reported in original publications.
	Family	Plant family according to World Flora Online 2023.
	Genus	Plant genus according to World Flora Online 2023.
	Species WFO	Plant species name according to World Flora Online 2023.
	Author WFO	Author of species according to World Flora Online 2023.
	Additional taxon	Additional information on taxon (cultivar [‘x’] or genotype [‘x’]), if given.
	Cultivation status	Whether the plant species is a wild or cultivated individual, according to the original publication. For studies not addressing the cultivation status of the plants, information on the species cultivation status was assessed from open sources.
Plant growth conditions	Country	The country where the study was conducted. If no country was mentioned in the methods, country of first authors institutional affiliation was used.
	Growth environment	Environment in which the plants producing the sampled pollen were grown, as reported in the original study (*field*, *glasshouse*, *growth chamber*, *both*, or *cut flowers*). *Field* refers to plants growing outdoors (wild populations, orchards, experimental plots, or botanical garden plantings). *Cut flowers* refers to studies using detached flowers for laboratory experiments where the original growing environment was not specified. For studies on tree pollen where cultivation conditions were not reported, the environment was classified as *field* by default.
Pollen viability estimation	Approach	The main approach used in the given study to assess pollen viability: germinability *in vitro*, stainability or stigmatic germinability.
	Method	Specific method or stain employed to test pollen viability; references to the original protocol are provided when reported in the study.
	Pollen medium ingredients	When the variable *Approach* was ‘*germinability in vitro*’, ingredients and pH (if reported) were listed, in the same order for clarity: agar (or alternative), sucrose, Ca(NO_3_)_2_, CaCl_2_, H_3_BO_3_, KNO_3_, MgSO_4_, other components, and pH.
Experimental conditions	Pretreatment	Pretreatments applied to pollen after collection but before storage (e.g. drying, desiccation, freeze‐drying).
	Storage conditions	Information on pollen storage conditions during the experiment, if provided (e.g. desiccator, liquid nitrogen).
	Storage relative humidity	Relative humidity (RH, %) within the storage container at the specified time point.
	Storage temperature	Temperature at which the pollen was stored (°C).
	Pollen moisture content	Pollen grain moisture content (%) at the given time point.
Pollen viability data	Time	Reported storage time at which pollen viability was assessed.
	Time unit	Time units as reported in the original sources: min = minutes, d = days, w = weeks, m = months, y = years.
	Time in days	Reported storage duration for the viability estimate, standardized to days to enable comparison across studies.
	Pollen viability	Estimated proportion of viable pollen (%) at the given time.
	Sample size	Number of pollen grains used in the study to assess *pollen viability*. Reported either as the total number of pollen grains or as replicates and pollen grains per replicate. Where the number of replicates was provided, but the number of pollen grains was not, the latter is denoted as ‘x’ (e.g., 3*x).
	Data extraction type	This variable specifies whether the value was read and extracted from the text in a table or estimated from a figure.
	SE	Standard error, if available, for the values in the ‘Pollen viability’ variable (reported in intervals of 5; both text‐read and figure‐estimated values included).
	SD	Standard deviation, if available, for the values in the ‘Pollen viability’ variable (reported in five intervals; both text‐read and figure‐estimated values included).
Pollen morphological traits (Extracted from external sources)	Pollen dispersal unit	The morphological unit of the pollen at the time of dispersal.
	Pollen size	The size categories of the pollen unit given by Halbritter *et al*. (2018), very small (< 10 μm), small (10–25 μm), medium (26–50 μm), large (51–100 μm), and very large (> 100 μm). For genus‐level information, some entries include two size categories.
	Aperture number	The number of apertures on the pollen grain.
	Nuclei number	The number of nuclei in the pollen grain of the given species from Paldat (2000), Brewbaker (1967), Williams *et al*. (2014) and Grayum (1986).
	Pollen morphology source	Source of pollen morphology data (i.e. *Pollen dispersal unit*, *Pollen size* and *Aperture number*). If the information is provided at the genus level, this is indicated before the reference.
Bibliography	Reference	Citation of the original source, including author(s) and year.
	Full citation	Complete reference formatted according to APA 6^th^ edition guidelines.
	Link	URL linking to the original article.

### 2. Data cleaning

All datapoints from the extracted articles were standardized if needed to ensure uniformity across the dataset: storage temperatures, where not specifically defined in the articles, were assigned standard values based on given wording (‘room temperature’ as 22°C and ‘liquid nitrogen’ as −196°C; Biopharma Group, 2025; Kiani, 2025); temperatures described in intervals or as fluctuations (e.g. 18–22°C) were presented as mean values; and original species names were harmonized against the World Flora Online 2023 (WFO, 2023), while the original species names were retained in the dataset.

### 3. Additional pollen data

Because pollen morphology and anatomical traits are correlated with pollen longevity (Dafni & Firmage, 2000; Pacini & Dolferus, 2019; Althiab‐Almasaud *et al*., 2023), we added such information to the dataset after data extraction. Specifically, we extracted information on pollen dispersal unit, pollen size, and number of apertures primarily from the Paldat (2000) database, relying on the species name in the publication. If the standardized species name was missing from Paldat, the name was cross‐checked in World Flora Online to identify relevant synonyms. If species‐level information was unavailable in PalDat, we provided data at the genus level and recorded it, as these traits are known to be strongly constrained by phylogeny (Erdtman, 1986; Taia, 2022). If the genus was not represented in the database, the data were retrieved from published literature. Pollen nuclei data were included primarily from Brewbaker (1967); in addition, we used Williams *et al*. (2014) and Grayum (1986).

## IV. Dataset structure

The structure of the pollen longevity and viability dataset (Dataset S1) encompasses seven data categories, six extracted from source publications, and one data category extracted from external sources (as explained above): taxonomy, plant growth conditions, pollen viability estimation, experimental conditions, pollen viability data, pollen morphological traits, and bibliography, with hierarchical variables visualized in Fig. 2 and further described in Table 1.

The shiny app (https://w1nther.shinyapps.io/Pollen‐longevity/) allows for data visualization of pollen viability over time, filtered by family, genus, species or reference. Within this filter, it is possible to further explore the experimental approach, sorting for filter and/or method. The filtered data can be downloaded as a .csv file for exploration across all variables.

## V. Current state of the dataset and implications for inference

The pollen longevity and viability dataset synthesizes data generated over more than six decades using diverse experimental designs, taxa, and pollen viability estimation approaches. While this breadth substantially expands data availability, it also imposes clear constraints on data integration and inference.

### 1. Taxonomic and cultivation bias

Our community resource currently comprises 466 species across 243 genera and 87 families, representing only a small fraction of global angiosperm diversity and including relatively few species of conservation concern. Consequently, the dataset provides limited coverage across plant diversity and cannot directly inform broad conservation assessments. Representation is highly uneven, with 33 families represented by only a single species and 60 families by fewer than five species. By contrast, Poaceae (52 species), Fabaceae (44 species), and Rosaceae (38 species) together account for a substantial proportion of the dataset. This imbalance is further amplified at the accession level: inclusion of cultivars and genotypes results in 1042 accessions, of which 886 are classified as cultivated and only 170 as wild, with just seven species represented in both categories. This pronounced skew reflects long‐standing applied research priorities focused on economically important crops and horticultural taxa rather than systematic ecological or phylogenetic coverage (Hanna & Towill, 1995; Barnabás & Kovács, 1997). Consequently, the ability to generalize patterns across different evolutionary and ecological scales is restricted. A limitation increasingly emphasized by recent conservation perspectives is the lack of pollen banking practices and protocols for wild species, thereby calling for targeted research into wild species and underrepresented families (Althiab‐Almasaud *et al*., 2023; Wolkis *et al*., 2024). As a result, the dataset is better suited for comparative analyses within well‐studied clades and cultivated systems than for phylogenetically comprehensive or macroecological generalizations across vascular plants. Broad evolutionary inference therefore requires explicit consideration of sampling bias and, where appropriate, the use of phylogenetically informed or hierarchical analytical frameworks (e.g. phylogenetic generalized least squares, mixed‐effects models, or Bayesian hierarchical approaches). A further limitation is that voucher information was only reported in a very small number of publications, preventing independent verification of taxonomic identities. Although this constraint does not affect the viability measurements, it limits long‐term traceability, underscoring the importance of including voucher details in future pollen research.

### 2. Temperature and storage conditions

**Fig. 3:**
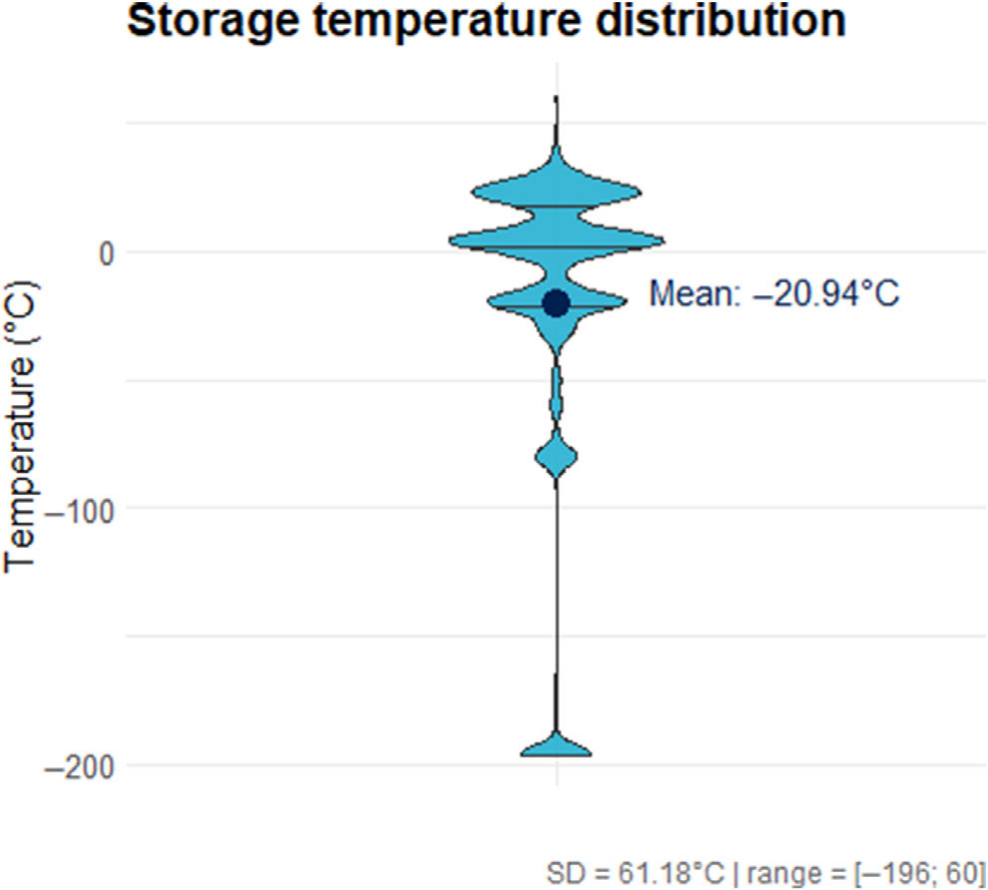
Distribution of storage temperature across all datapoints in the dataset (°C). Lines in the violin plot mark quartiles, with a point marking the mean. The data range from −196 to 60°C (SD = 61.18).

The dataset spans an exceptionally wide range of storage temperatures (Fig. 3), from cryogenic storage at −196°C to short‐term storage at 60°C (Sedgley & Harbard, 1993). Of the 320 publications included, 130 assess pollen longevity at ultra‐low temperatures of −50°C or lower, 180 at freezing temperatures (−50 to 0°C), 187 at cold storage above freezing (0 to 10°C), and 177 include temperatures above 10°C. Many studies assess multiple temperature regimes to demonstrate the extension of pollen longevity at lower temperatures. Overall, the mean storage temperature across the dataset is −20.9°C, emphasizing the applied focus on pollen banking, germplasm exchange, and breeding, rather than the assessment of pollen longevity under natural or near‐natural environmental conditions (Dafni & Firmage, 2000; Althiab‐Almasaud *et al*., 2023; Stokes & Geitmann, 2025). Moreover, key water‐related variables known to interact with temperature, such as pollen moisture content, storage relative humidity, and rehydration protocols, are inconsistently reported across studies, limiting direct comparability among experiments. Necessary standardization of ambiguous descriptors (e.g. ‘room temperature’ or ‘liquid nitrogen’) introduces additional uncertainty into cross‐study comparisons. Accordingly, the dataset is not designed to predict absolute pollen longevity in ecological field contexts, but rather to support relative, comparative analyses across controlled storage regimes.

### 3. Temporal distribution of studies

The publications included in the database span from 1962 to 2023, from early observational studies of pollen viability to modern experimental research on long‐term pollen storage under ultra‐low temperatures, reflecting the progressive development of experimental approaches in this field. Earlier studies often relied on simpler experimental designs and reported fewer methodological details, whereas more recent publications typically employ more standardized protocols and provide more extensive metadata. Such differences may influence the comparability of reported pollen viability values across studies. However, because the database is primarily intended to support comparative analyses within defined experimental contexts rather than to estimate absolute pollen lifespan, variation associated with publication date is expected to have limited influence when methodological differences are explicitly considered in analyses.

### 4. Methodological bias in pollen viability estimation

**Fig. 4:**
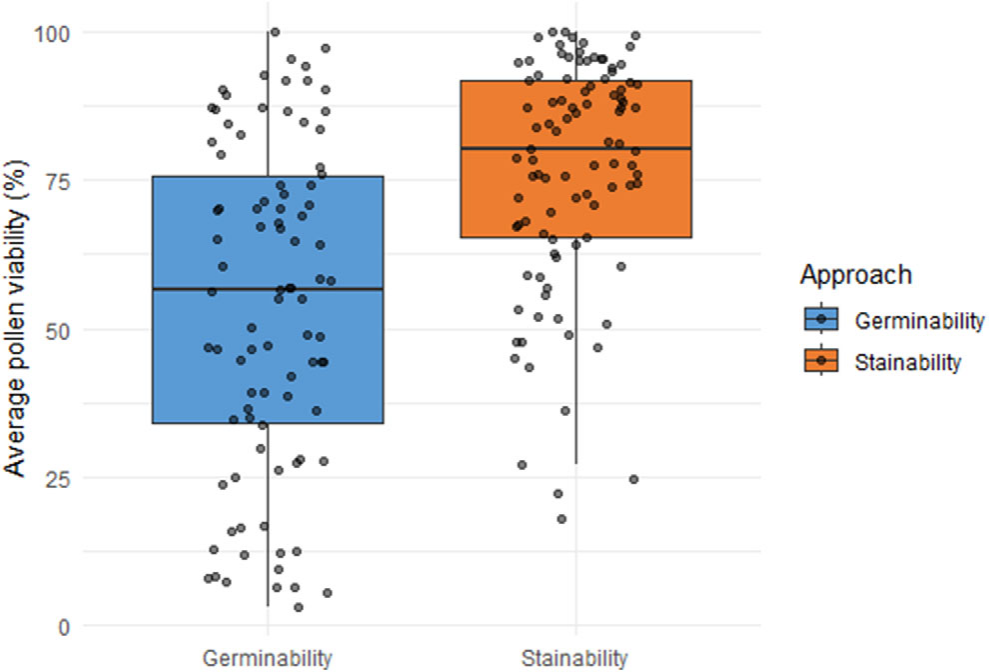
Differences in pollen initial viability (time = 0) according to estimation approach (pollen germinability or stainability). Each point represents a species in the references including both approaches. Boxplots show the median, with whiskers extending 1.5 times the interquartile range.

Pollen longevity can be estimated in several ways, with the most common approaches being *in vitro* germination assays or staining techniques. In this dataset, 68% of publications estimate longevity using *in vitro* germination assays, 11% rely exclusively on staining, 20% report both approaches, and only 1% assess stigmatic germinability. Where both staining and germination are reported within the same study, initial viability estimates are on average higher for staining (80%) than for *in vitro* germination (59%; Fig. 4), consistent with well‐established methodological biases whereby staining can overestimate viability and germination assays may underestimate it (Dafni & Firmage, 2000). The most frequently used staining methods were FCR (38 studies), TTC (23), and acetocarmine (20), each targeting different cellular components. For *in vitro* germination, many studies draw on the medium by Brewbaker & Kwack (1963), a widely accepted baseline for angiosperm pollen. Because these methods capture distinct physiological processes, viability estimates derived from different approaches are not directly interchangeable. Analyses that pool data across methods without accounting for this heterogeneity risk generate artefactual patterns. We therefore encourage the combined use of *in vitro* germination and staining to obtain a more coherent estimate of viability, along with reporting media composition and experimental conditions to enable replication and cross‐study synthesis. The dataset is best suited for method‐aware comparative analyses, in which the viability estimation approach is treated explicitly as an explanatory or stratifying factor (e.g. as a fixed effect, random effect, or grouping variable in comparative models), rather than as measurement noise.

## VI. Opportunities for synthesis and application of pollen longevity data

This dataset provides a framework and starting point for comparative research on pollen viability and longevity, integrating data across taxa, storage regimes, and experimental approaches. By consolidating information that was previously scattered across disciplines, languages, and publication formats, it enables analyses that extend beyond single‐species or single‐study perspectives.

At a fundamental level, the dataset supports phylogenetically informed comparative analyses of pollen longevity across species differing in ecology, reproductive biology, and pollen structure and performance within the currently represented taxa and experimental systems. Potentially in combination with other data, this dataset enables tests of how pollen longevity relates to species ecology, plant biological traits and physiological characteristics and whether a combination of these traits forms distinct pollen longevity syndromes. Beyond trait associations, the resource allows examination of trade‐offs between pollen longevity and reproductive strategies. Together, these opportunities connect pollen longevity to broader theories of reproductive assurance, mating system evolution, and pollination ecology, advancing its interpretation as a functional trait shaped by evolutionary history, ecological context, and reproductive strategy.

The extensive coverage of storage temperatures, experimental conditions, and time points further enables analyses of pollen viability decay under controlled environments. Rather than estimating absolute pollen lifespan, the data support comparative assessments of relative sensitivity to temperature and storage duration among taxa, functional groups, or pollen trait categories, informing hypotheses about stress resistance and cellular stability. These insights also have clear applied relevance: comparative analyses may help identify species or groups with inherently short pollen longevity among the taxa represented in the dataset, which could be particularly vulnerable to reproductive failure in fragmented landscapes or under climate stress. In conservation and restoration, such information can inform the timing of pollen collection, storage, and assisted pollination, while in crop improvement and germplasm conservation, it provides a comparative framework for evaluating pollen storage performance across taxa, reducing reliance on species‐specific trial‐and‐error approaches.

Finally, the representation of multiple pollen viability estimation approaches enables explicit assessment of methodological effects on inferred pollen longevity. Comparative analyses can evaluate the consistency of viability decay trajectories across methods, quantify systematic differences between staining‐ and germination‐based estimates, and assess how methodological choice influences comparative conclusions. Together, these opportunities position the dataset as a platform for integrative research within the currently represented taxa while highlighting important knowledge gaps across plant diversity. The current database represents an initial synthesis of published pollen longevity data and provides a framework that can be expanded as additional studies and taxa are incorporated in future updates.

## Competing interests

None declared.

## Author contributions

SR conceived the idea. LW and SR constructed the dataset and wrote the manuscript. LW performed the literature search and data extraction and led the writing of the manuscript. LW and SR created the Shiny App with support from Copilot. CBAL contributed to manuscript writing and revision. All authors contributed to writing and approved the final version of the manuscript.

## Disclaimer

The New Phytologist Foundation remains neutral with regard to jurisdictional claims in maps and in any institutional affiliations.

## Supporting information


**Dataset S1** Pollen longevity and viability dataset.

**Fig. S1** PRISMA 2020 flow diagram of literature search.
**Notes S1** References of primary sources included in dataset.
Please note: Wiley is not responsible for the content or functionality of any Supporting Information supplied by the authors. Any queries (other than missing material) should be directed to the *New Phytologist* Central Office.

## Data Availability

The pollen longevity and viability dataset is openly available as Dataset S1 and via https://doi.org/10.5281/zenodo.19330491. An interactive Shiny App is accessible at https://w1nther.shinyapps.io/Pollen‐longevity/, allowing users to explore, filter, visualize, and download customized subsets of the data. All data were extracted from published primary sources, which are cited in the dataset and Notes S1.
