# Supplementary material for: Pollen longevity and viability dataset: an integrated resource for plant research and conservation
Source: New Phytol. 2026 Jun 2;251(4):1631–9. doi: 10.1111/nph.71325 (PMC13373826; doi:10.1111/nph.71325)
Supplement: Supplementary file 2 — Fig. S1 PRISMA 2020 flow diagram of literature search. Notes S1 References of primary sources included in dataset. Please note: Wiley is not responsible for the content or functionality of any Supporting Information supplied by the authors. Any queries (other than missing material) should be directed to the New Phytologist Central Office. [file NPH-251-1631-s001.pdf]

New Phytologist

***New Phytologist* Supporting Information**

Article title: Pollen longevity and viability dataset: an integrated resource for plant research and conservation
Authors: Louise Winther, Conny Bruun Asmussen Lange, Sergey Rosbakh
Article acceptance date: 18 May 2026

The following Supporting Information is available for this article:

Fig. S1 PRISMA 2020 flow diagram of literature search

Notes S1 References of primary sources included in dataset

Dataset S1 Pollen longevity dataset (separate file)

Fig. S1 PRISMA 2020 flow diagram by Page *et al.* (2021), modified and filled to illustrate the literature search for data extraction to the Pollen longevity dataset.

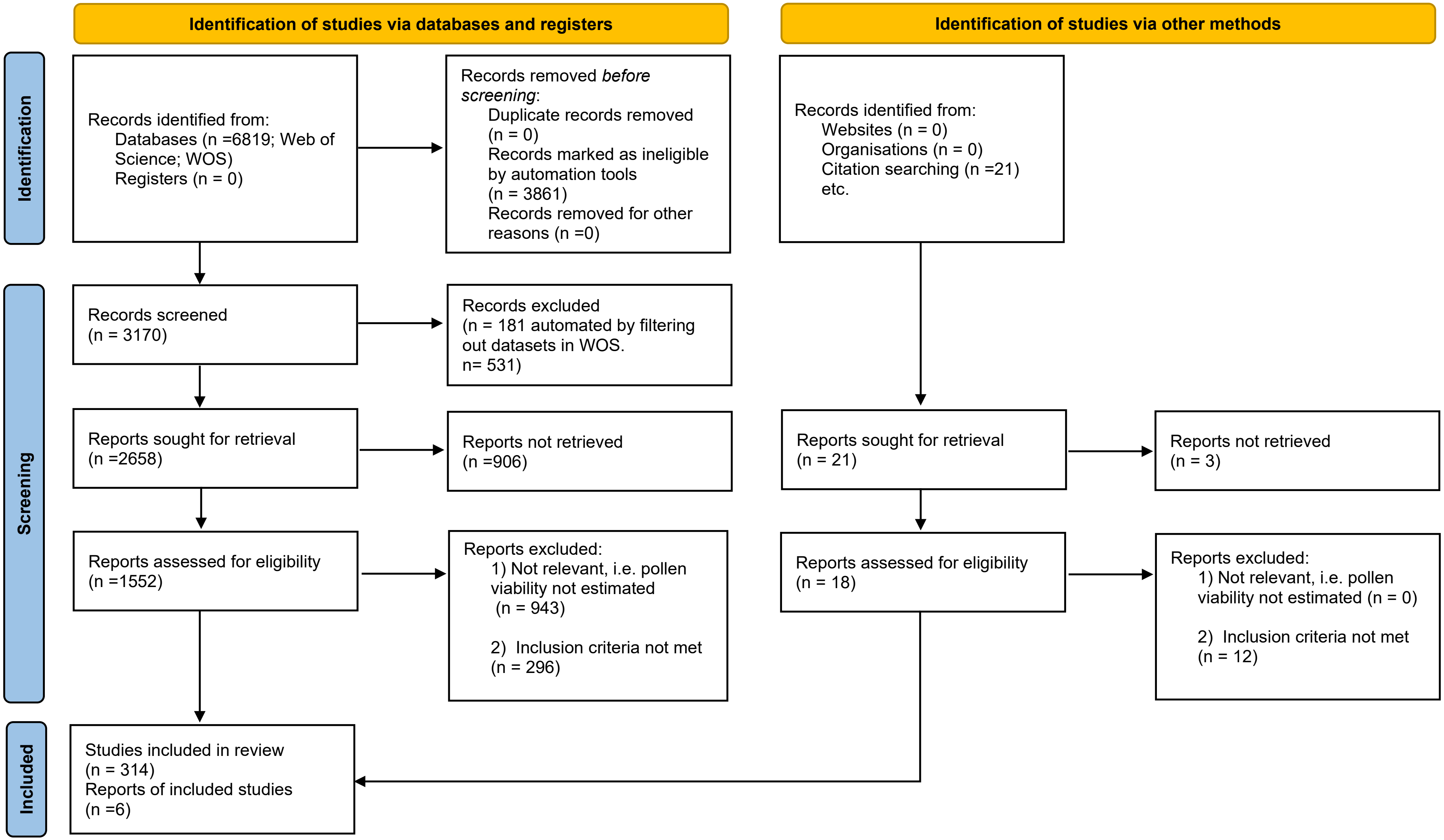

Notes S1 References of primary sources included in dataset including references from sources of pollen morphology

Abdul-Baki AA. 1992. Determination of pollen viability in tomatoes. *Journal of the American Society for Horticultural Science* **117**(3): 473-476.

Adaniya S, Higa T. 1988. Effects of temperature and relative humidity on pollen germinability and Pollen Tube Growth in the Style of *Zingiber mioga* Roscoe. *Journal of the Japanese Society for Horticultural Science* **57**(1): 43-51.

Afshari H, Talaei A, Panahi B, Hokmabadi H. 2008. Morphological and qualitative study of pistachio *(Pistacia vera L.)* pollen grains and effect of different temperatures on pomological traits. *Australian Journal of Crop Science* **1**(3): 108-114.

Agueguia A, Fatokun CA. 1988. Pollen storage in cocoyam *(Xanthosoma sagittifolium* (L.) Schott). *Euphytica* **39**(3): 195-198.

Ahi Kosar D, Erturk U. 2023. Effects of Caprifig (Ficus carica var. caprificus) Storage Temperature and Duration on the Fruit Productivity and Quality of 'Bursa Siyahi' Figs. *Horticulturae* **9**(1): 78.

Akihama T, Omura M, Kozaki I. 1979. Long-term Storage of Fruit Tree Pollen and Its Application in Breeding. *JARQ - Japan Agricultural Research Quarterly* **13**(4): 238-241.

Akond ASMGM, Pounders CT, Blythe EK, Wang X. 2012. Longevity of crapemyrtle pollen stored at different temperatures. *Scientia horticulturae* **139**: 53-57.

Akoroda MO. 1983. Long-term storage of yam pollen. *Scientia horticulturae* **20**(3): 225-230.

Al-Najm A, Brauer S, Trethowan R, Merchant A, Ahmad N. 2021. Optimization of in vitro pollen germination and viability testing of some Australian selections of date palm (*Phoenix dactylifera* L.) and their xenic and metaxenic effects on the tissue culture-derived female cultivar "Barhee". *In Vitro Cellular & Developmental Biology-Plant* **57**(5): 771-785.

Alburquerque N, Garcia Montiel F, Burgos L. 2007. Short communication. Influence of storage temperature on the viability of sweet cherry pollen. *Spanish Journal of Agricultural Research* **5**(1): 86-90.

Aldahadha A, Al Sane K, Bataineh A, Abu Alloush A, Hamouri Z. 2019. Pollen viability and in vitro germination of six pistachio (*Pistacia vera* L.) cultivars grown in northern Jordan. *Advances in Horticultural Science* **33**(3): 441-446.

Ali S, Perveen A. 2021. Pollen vitality and germination capacity in three taxa of the genus *Brassica* L. (Brassicaceae). *Pakistan Journal of Botany* **53**(3): 1079-1082.

Almeida NVd, Saziki CYN, Cardoso JC. 2019. Characterization of cultivars and low-temperature pollen grain storage in amaryllis (*Hippeastrum sp*.). *Revista Ceres* **66**(6): 451-459.

Alzer FdC, Couto R, Gonçalves-Esteves V, Mendonça CBF. 2024. Pollen variability in species of *Dioscorea* L. (Dioscoreaceae). *Grana* **63**(1).

Amma S, Watanabe A. 1985. Long-Term Storage of Germ Plasm of Tea (*Camellia sinensis* (L.) O.Kuntze). *Jarq* **19**(3): 196-201.

Araújo D, Chagas P, Chagas E, Moura E, de Oliveira R, Taveira D, Ribeiro M, Grigio M. 2021. Flower stages, germination and viability of pollen grains of *Annona squamosa* L. in tropical conditions. *Acta Scientiarum. Technology* **43**.

Araujo de Oliveira AC, Ledo AdS, Polek M, Krueger R, Shepherd A, Volk GM. 2021. Optimization of in vitro germination and cryopreservation conditions for preserving date palm pollen in the USDA National Plant Germplasm System. *Plant Cell Tissue and Organ Culture* **144**(1): 223-232.

Aslantas R, Pirlak L. 2001. Storage of strawberry pollen. *Acta Agrobotanica* **54**(2): 117-121.

Ateyyeh AF. 2009. Storing Pollen of Two Olive Cultivars: 'Rasie' and 'Nabali Baladi'. *International Journal of Fruit Science* **9**(3): 282-293.

Baez P, Riveros M, Lehnebach C. 2002. Viability and longevity of pollen of *Nothofagus* species in south Chile. *New Zealand Journal of Botany* **40**(4): 671-678.

Bahramabadi EZ, Jonoubi P, Rezanejad F. 2018. Ultrastructural changes of pistachio (*Pistacia vera* L.) mature seeds and pollen in relation to desiccation. *Trees-Structure and Function* **32**(1): 29-39.

Bai W-N, Zeng Y-F, Liao W-J, Zhang D-Y. 2006. Flowering phenology and wind-pollination efficacy of heterodichogamous *Juglans mandshurica* (Juglandaceae). *Annals of botany* **98**(2): 397-402.

Bal JS, Nandan R 2012. Pollen Morphology of Some Selected Plum Genotypes in the Sub-Tropics. *10th International Symposium on Plum and Prune Genetics, Breeding and Pomology*. Davis, CA. 57-63.

Barbieri CR, Nava GA. 2020. Production and in vitro viability of pollen of peach trees grown in subtropical climate. *Revista Brasileira De Fruticultura* **42**(3).

Barbosa W, Campo-Dall'Orto FA, Ojima M, Martins FP, Boaventura YMS. 1991. Conservação e germinação do pólen, polinização e frutificação efetiva em pessegueiros e nectarineiras subtropicais. *Bragantia* **50**(1): 17-28.

Barnabás B, Rajki E. 1976. Storage of maize (*Zea mays* L.) pollen at −196°C in liquid nitrogen. *Euphytica* **25**(1): 747-752.

Bassani M, Pacini E, Franchi GG. 1994. Humidity stress responses in pollen of anemophilous and entomophilous species. *Grana* **33**(3): 146-150.

Batos B, Miljkovic D. 2019. The vitality of the Serbian spruce (*Picea omorika*) pollen during the long-term cryopreservation. *Grana* **58**(6): 433-446.

Batos B, Miljkovic D, Bobinac M. 2012. Some characters of the pollen of spring and summer flower Common Oak (*Quercus robu*r L.). *Archives of Biological Sciences* **64**(1): 85-95.

Batos BZ, Nikolic BM. 2013. Variability of in vitro germination of *Picea omorika* pollen. *Dendrobiology* **69**: 13-19.

Bellani LM, Forino LMC, Tagliasacchi AM, Sansavini S. 1984. Viability of Stored Pollen Grains of *Malus domestica* Borkh as Evaluated by the Incorporation of Labelled Nucleic Acid Precursors. *Caryologia* **37**(4): 323-330.

Bocquel C. 1995. Technology and preservation and storage birch pollen (*Betula verrucosa* Ehrh). In vitro germination of native and polluted pollen: Influence of environmental factors. *Grana* **34**(6): 413-420.

Bogner J, Gonçalves EG. 2005. Two new species of *Xanthosoma* (Araceae) from South America and notes on the tribe Caladieae. *Willdenowia* **35**(2): 333-344.

Bomben C, Malossini C, Cipriani G, Testolin R 1999. Long term storage of kiwifruit pollen. *4th International Symposium on Kiwifruit*. Santiago, Chile. 105-108.

Borghezan M, Clauman AD, Steinmacher DA, Guerra MP, Orth AI. 2011. In vitro viability and preservation of pollen grain of kiwi (*Actinidia chinensis* var. *deliciosa* (A. Chev.) A. Chev). *Crop Breeding and Applied Biotechnology* **11**(4): 338-344.

Bowes SA. 1990. Long-term storage of *Narcissus* anthers and pollen in liquid nitrogen. *Euphytica* **48**(3): 275-278.

Broglia M, Brunori A. 1994. Synergistic Effect of Low Temperature and High Sucrose Concentration on Maize Pollen Viability in Aqueous Medium. *Crop science* **34**(2): 528-529.
Brown MJ. 1997. *Durio: A bibliographic review*. New Dehli, India: IPGRI office for South Asia.
Buchner L, Eisen A-K, Sikoparija B, Jochner-Oette S. 2022. Pollen Viability of Fraxinus excelsior in Storage Experiments and Investigations on the Potential Effect of Long-Range Transport. *Forests* **13**(4).
Calic D, Milojevic J, Belic M, Miletic R, Zdravkovic-Korac S. 2021. Impact of Storage Temperature on Pollen Viability and Germinability of Four Serbian Autochthon Apple Cultivars. *Frontiers in plant science* **12**.
Candido RS, Fourny ACdS, Gonçalves-Esteves V, Lopes RC. 2013. *Hippeastrum* species in areas of restinga in the state of Rio de Janeiro, Brazil: pollen characters. *Acta Botanica Brasilica* **27**(4).
Cao M, Zhang D-X, Shah A, Dong L. 2014. Pollen morphology and its systematic significance in *Zanthoxylum* (Rutaceae) from China. *Pakistan Journal of Botany* **46**(4): 1325-1330.
Carreno J, Oncina R, Carreno I 2009. In Vitro Studies on Pollen Germination Capability and Preservation of Different Cultivars of *Vitis vinifera* L. *9th International Conference on Grape Genetics and Breeding*. Udine, ITALY. 493-496.
Cauneau-Pigot A. 1988. Biopalynological Study of *Lapageria rosea* and *Iris unguicularis*: Storage of Pollen. *Grana* **27**(4): 297-312.
Chanda SC, Sagar A, Islam MM, Hossain MA, Sarwar AKMG. 2019. Phenology and reproductive biology of three Sesbania species. *International Journal of Minor Fruits, Medicinal and Aromatic Plants* **5**(1): 29-37.
Chander S, Rajasekhara PE, Kurian RM. 2019. Pollen storage studies in sugar apple (*Annona squamosa* L.) cv. Balanagar. *Israel Journal of Plant Sciences* **66**(3-4): 196-202.
Chang WN, Struckmeyer BE. 1975. The Influence of Temperature and Relative Humidity on Onion Pollen Germination. *Hortscience* **10**(2): 162-163.
Chaudhury R, Malik SK, Rajan S. 2010. An Improved Pollen Collection and Cryopreservation Method for Highly Recalcitrant Tropical Fruit Species of Mango (*Mangifera indica* L.) and Litchi (*Litchi chinensis* Sonn.). *Cryoletters* **31**(3): 268-278.
Chiang MS. 1974. Cabbage pollen germination and longevity. *Euphytica* **23**(3): 579-584.
Ching TM, Ching KK. 1964. Freeze-Drying Pine Pollen. *Plant Physiology* **39**(5): 705-709.
CIQTEK. 2022. *Explore Pollen Micromorphology - Scanning Electron Microscope (SEM) Applications*. https://www.ciqtekglobal.com/explore-pollen-micromorphology-scanning-electron-microscope-sem-applications_n44.
Cohen E, Lavi U, Spiegelroy P. 1989. Papaya pollen viability and storage. *Scientia horticulturae* **40**(4): 317-324.
Collins FC, Lertmongkol V, Jones JP. 1973. Pollen Storage of Certain Agronomic Species in Liquid Air. *Crop science* **13**(4): 493-494.
Cram WH, Lindquist CH. 1984. Pollen Viability Studies for *Picea pungens*. *The Forestry Chronicle* **60**(2): 93-95.
Cruzatty LCG, Droppelmann F, Izaguirre-Mayoral ML. 2020. New protocol for storage of viable pollen of *Lapageria rosea* (Philesiaceae), an endangered plant species endemic to temperate forests of Chile. *Plant Species Biology* **35**(4): 332-337.
Cuchiara CC, Silva SDDAE, Bobrowski VL. 2012. Conservação de grãos de pólen de mamoneira a baixas temperaturas. *Revista Ceres* **59**(1): 82-87.
Custodio CC, Machado-Neto NB, Singer RB, Pritchard HW, Seaton PT, Marks TR. 2020. Storage of orchid pollinia with varying lipid thermal fingerprints. *Protoplasma* **257**(5): 1401-1413.

Custodio L, Romano A, Fernandes N, Carneiro MF 2004. Cryopreservation of pollen of carob tree. *5th International Symposium on In Vitro Culture and Horticultural Breeding*. Debrecen, HUNGARY. 863-+.

D'Antonio V, Quiros CF. 1987. Viability of Celery Pollen After Collection and Storage. *Hortscience* **22**(3): 479-481.

da Silva RL, de Souza EH, Vieira LdJ, Pelacani CR, Duarte Souza FV. 2017. Cryopreservation of pollen of wild pineapple accessions. *Scientia horticulturae* **219**: 326-334.

Daher FB, Chebli Y, Geitmann A. 2009. Optimization of conditions for germination of cold-stored *Arabidopsis thaliana* pollen. *Plant Cell Reports* **28**(3): 347-357.

Daniel IO. 2011. Exploring storage protocols for yam (*Dioscorea spp.*) pollen genebanking. *African Journal of Biotechnology* **10**(42): 8306-8311.

Daniel IO, Ng NQ, Tayo TO, Togun AO. 2002. Wet-cold preservation of West African yam (*Dioscorea spp.*) pollen. *Journal of Agricultural Science* **138**: 57-62.

Danner MA, Citadin I, Sasso SAZ, Sachet MR, Malagi G. 2011. Modo de reprodução e viabilidade de pólen de três espécies de jabuticabeira. *Revista Brasileira De Fruticultura* **33**(2): 345-352.

Davarynejad G, Rashed M, Vatanpoor A, Csillag F 1995. The morphology of pollen grains as an indicator for identification of male pistachio (*Pistacia vera* L.) trees. *International Symposium on Pistachio*. 37-42.

De Leonardis W, Fichera G, Ocampo B, Venora G, Vona S, Zizza A. 1995. Correlation between pollen grain and seed size in *Cicer* species. *Journal of Genetics and Breeding* **49**: 21-21.

Dehgan B, Dehgan NB. 1988. Comparative pollen morphology and taxonomic affinities in Cycadales. *American Journal of Botany* **75**(10).

Demchik SM, Day TA. 1996. Effect of enhanced UV-B radiation on pollen quantity, quality, and seed yield in *Brassica rapa* (Brassicaceae). *American Journal of Botany* **83**(5): 573-579.

Demeke T, Hughes HG. 1991. Germination and Storage of Pollen of Phytolacca dodecandra L. (endod). *Annals of botany* **68**(1).

Deng ZN, Harbaugh BK. 2004. Technique for in vitro pollen germination and short-term pollen storage in *Caladium*. *Hortscience* **39**(2): 365-367.

Devi I, Singh H, Thakur A, Singh J. 2018. Optimization of pollen storage conditions for low chill peach cultivars. *Indian Journal of Horticulture* **75**(4): 560-566.

Dimitruk M. 2012. Biology of flowering and nectar production in the flowers of the beauty bush (*Kolkwitzia amabilis* Graebn.). *Acta Agrobotanica* **65**(4).

Dinato NB, Imaculada Santos IR, Leonardecz E, Burson BL, Quarin CL, de Paula AF, Favero AP. 2018. Storage of Bahiagrass Pollen at Different Temperatures. *Crop science* **58**(6): 2391-2398.

Dordevic M, Vujovic T, Cerovic R, Glisic I, Milosevic N, Maric S, Radicevic S, Aksic MF, Meland M. 2022. In Vitro and In Vivo Performance of Plum (*Prunus domestica* L.) Pollen from the Anthers Stored at Distinct Temperatures for Different Periods. *Horticulturae* **8**(7).

Dutta SK, Srivastav M, Chaudhary R, Lal K, Patil P, Singh SK, Singh AK. 2013. Low temperature storage of mango (*Mangifera indica* L.) pollen. *Scientia horticulturae* **161**: 193-197.

Ecker G, Meyer T, Auer C. 2013. Pollen Longevity and Dispersion Models for Switchgrass. *Crop science* **53**(3): 1120-1127.

Eenink AH. 1983. Preliminary results of research on storage and in vitro germination of lettuce pollen as an aid in lettuce breeding. *Euphytica* **32**(2): 521-526.

Ekaratne SNR, Senathirajah S. 1983. Viability and Storage of Pollen of the Oil Palm *Elaeis guineensis* Jacq. *Annals of botany* **51**(5): 661-668.

El-Amier YA. 2015. Morphological studies of the pollen grains for some hydrophytes in coastal Mediterranean lakes, Egypt. *Egyptian Journal of Basic and Applied Sciences* **2**(2).

El Naggar S, Sawady N. 2008. Pollen morphology of Malvaceae and its taxonomic significance in Yemen. *Flora Mediterranea* **18**: 431-439.

Elansary M, Winkelmann T. 2014. Preparing Interspecific Hybridization in the Genus *Lobelia* - Nuclear DNA Contents, Pollen Handling, and Localization of Crossing Barriers. *European Journal of Horticultural Science* **79**(6): 290-299.

Farcy E, Verhille A-M, Cornu A, Cerceau-Larrival M-T. 1990. Etude de la conservation du pollen de Pétunia: méthodologie, tests de viabilité, (in vitro et en vivo), et de conformité génétique. *Bulletin de la Société Botanique de France. Actualités Botaniques* **137**(2): 105-110.

Farmer RE, Barnett PE. 1974. Low-temperature storage of black walnut pollen. *Cryobiology* **11**(4): 366-367.

Fayos O, Echavarri B, Pilar Valles M, Mallor C, Garces-Claver A, Castillo AM. 2022. A simple and efficient method for onion pollen preservation: Germination, dehydration, storage conditions, and seed production. *Scientia horticulturae* **305**.

Fei S, Nelson E. 2003. Estimation of pollen viability, shedding pattern, and longevity of creeping bentgrass on artificial media. *Crop science* **43**(6): 2177-2181.

Fernandez Pacella L, Di Pasquo M. 2022. Morfología del polen y esporas del Holoceno de los Esteros del Iberá en el noreste de Argentina. *Boletín de la Sociedad Argentina de Botánica* **57**(4).

Fernando DD, Richards JL, Kikkert JR. 2006. In vitro germination and transient GFP expression of American chestnut (*Castanea dentata*) pollen. *Plant Cell Reports* **25**(5): 450-456.

Ferri A, Giordani E, Padula G, Bellini E. 2008. Viability and in vitro germinability of pollen grains of olive cultivars and advanced selections obtained in Italy. *Advances in Horticultural Science* **22**(2): 116-122.

Filho JGS, Torres AC, Nascimento WM, Boiteux LS 2012. Tomato hybrid seed production using stored pollen grains: temperature and storage time interactions and their effects on pollen viability. *6th International Symposium on Seed, Transplant and Stand Establishment of Horticultural Crops*. Brasilia, BRAZIL. 201-204.

Franzon RC, Raseira MDCB. 2006. Germinação in vitro e armazenamento do pólen de *Eugenia involucrata* DC (Myrtaceae). *Revista Brasileira De Fruticultura* **28**(1): 18-20.

Furness CA. 2012. Pollen Evolution in the Clusioid Clade (Malpighiales). *International Journal of Plant Sciences* **173**(9).

Furumoto R, Kato T. 2019. A simple way to store *Calophyllum inophyllum* L. pollen. *Bulletin of the Forestry and Forest Products Research Institute* **18**(1): 33-34.

Ganeshan S. 1985. Cryogenic preservation of grape (*Vitis vinifera* L.) pollen. *Vitis* **24**(3): 169-173.

Ganeshan S. 1986. Cryogenic preservation of papaya pollen. *Scientia horticulturae* **28**(1-2): 65-70.

Ganeshan S, Alexander M. 1990. Fertilizing ability of cryopreserved grape (*Vitis vinifera* L.) pollen. *Vitis* **29**(3): 145-150.

Gang Bangchu WJ. 1997. Study on pollination biology in persimmon trees. *Forest Research* **10**(3): 237-243.

Garcia Cruzatty L, Rivero M, Droppelmann F. 2015. Effect of temperature and drying on the longevity of stored *Nothofagus alpina* pollen. *New Zealand Journal of Botany* **53**(3): 155-164.

Garcia Talledo B, Bazurto Zambrano A, Garcia Cruzatty L, Zambrano Gavilanes F. 2019. Morphology, viability, and longevity of pollen of National Type and Trinitarian (CCN-51) clones of cocoa (*Theobroma cacao* L.) on the Coast of Ecuador. *Brazilian Journal of Botany* **42**(3): 441-448.

Gates P, Boulter D. 1980. The use of pollen Isoenzymes as an aid to the breeding of field beans (*Vicia faba* L.). *New Phytologist* **84**(3): 501-504.

Gaudet D, Yadav NS, Sorokin A, Bilichak A, Kovalchuk I. 2020. Development and Optimization of a Germination Assay and Long-Term Storage for *Cannabis sativa* Pollen. *Plants-Basel* **9**(5).

Gay G, Kerhoas C, Dumas C. 1987. Quality of a stress-sensitive *Cucurbita pepo* L. pollen. *Planta* **171**(1): 82-87.

Ge Y, Fu C, Bhandari H, Bouton J, Brummer EC, Wang Z-Y. 2011. Pollen Viability and Longevity of Switchgrass (*Panicum virgatum* L.). *Crop science* **51**(6): 2698-2705.

Geng X, Qiu J, Okubo H. 2013. Changes of carbohydrate content during *Lilium* and *Gladiolus* pollen cryopreservation. *Grana* **52**(3): 202-206.

Georgieva ID, Kruleva MM. 1993. Cytochemical investigation of long-term stored maize pollen. *Euphytica* **72**(1-2): 87-94.

Ghosh A, Karmakar P. 2017. Monocot pollen flora of Paschim Medinipur District, West Bengal with a note on pollen dispersal mechanism. *Current Botany* **8**.

Gimondo JA. 2022. Biotic and abiotic risks to *Dirca spp.* in managed landscapes. *(Doctoral dissertation, Iowa State University)*.

Giovannini A, Macovei A, Caser M, Mansuino A, Ghione GG, Savona M, Carbonera D, Scariot V, Balestrazzi A. 2017. Pollen Grain Preservation and Fertility in Valuable Commercial Rose Cultivars. *Plants-Basel* **6**(2).

Gomes PR, Raseira MDCB, Baudet LL, Peske ST. 2003. Armazenamento do grão de pólen de cebola (*Allium cepa* L.). *Revista Brasileira de Sementes* **25**(1): 14-17.

Guarnieri M, Speranza A, Nepi M, Artese D, Pacini E. 2006. Ripe pollen carbohydrate changes in *Trachycarpus fortunei*: the effect of relative humidity. *Sexual Plant Reproduction* **19**(3): 117-124.

Gunver-Dalkilic G, Dayi-Dogru O. 2011. Determination of pollen grain viability and germination levels for Pistachio and Terebinth in Aydin / Turkey ecology. *Pakistan Journal of Botany* **43**(2): 841-848.

Guo J, Dong X, Li Y, Wang B. 2020. NaCl treatment markedly enhanced pollen viability and pollen preservation time of euhalophyte Suaeda salsa via up regulation of pollen development-related genes. *Journal of Plant Research* **133**(1): 57-71.

Harley MM, Song U, Banks HI. 2005. Pollen morphology and systematics of Burseraceae. *Grana* **44**(4).

Hecker RJ, Stanwood PC, Soulis CA. 1986. Storage of sugarbeet pollen. *Euphytica* **35**(3): 777-783.

Hegde V, Koundinya AVV, Sheela MN, Chandra CV, Mukherjee A. 2019. Storage of cassava pollen for conservation of nuclear genetic diversity and overcoming hybridization barriers. *Indian Journal of Horticulture* **76**(1): 104.

Henny RJ. 1978. Germination of *Spathiphyllum* and *Vriesea* Pollen after Storage at Different Temperatures and Relative Humidities. *Hortscience* **13**(5): 596-597.

Herber BE. 2002. Pollen morphology of the Thymelaeaceae in relation to its taxonomy. *Plant Systematics and Evolution* **232**(1).

Heslop-Harrison JS 1992. Pollen Capture, Adhesion and Hydration. *Sexual Plant Reproduction*: Springer Berlin Heidelberg, 81-88.

Honda K, Watanabe H, Tsutsui K. 2002. Cryopreservation of *Delphinium* pollen at-30 degrees C. *Euphytica* **126**(3): 315-320.

Honsho C, Somsri S, Tetsumura T, Yamashita K, Yapwattanaphun C, Yonemori K. 2007. Characterization of male reproductive organs in durian; Anther dehiscence and pollen longevity. *Journal of the Japanese Society for Horticultural Science* **76**(2): 120-124.

Huang Pu-Hwa MJ-S. 1987. Studies on Pollen Morphology and Taxonomy of *Lespedeza* and Its Allied Genera from NE China. *Journal of Systematics and Evolution* **25**(5): 366-370.

Huang ZH, Zhu JM, Mu XJ, Lin JX. 2004. Pollen dispersion, pollen viability and pistil receptivity in *Leymus chinensis*. *Annals of botany* **93**(3): 295-301.

Husain F, Mallikarjuna N, Jadhav D. 2008. Pollen preservation and germination studies in *Arachis* species. *Indian Journal of Genetics and Plant Breeding* **68**(3): 334-336.

Hussain MM, Chakraborty P, Bhattacharya K, Hussain MM, Chakraborty P, Bhattacharya K. 2011. Pollen grains of queen sago (*Cycas circinalis* L.), a source of aeroallergen from West Bengal, India: an immunochemical approach. *Aerobiologia* **28**(1).

Haarring R, Zimmerman LH, Smith JD. 1969. Short and Long Period Storage of Pollen of *Ricinus communis* L. *Crop science* **9**(1): 17-+.

Iizuka M, Kudo N, Kimura Y, Ogiwara I. 2001. Interspecific hybrids between *Spiraea thunbergii* Sieb. ex Blume. and *S. japonica* L. fil. via ovule culture. *Journal of the Japanese Society for Horticultural Science* **70**(6): 767-773.

Ikeda H, Numata S 1996. Pollen storage of chrysanthemum. *3rd International Symposium on New Floricultural Crops*. Perth, Australia. 329-333.

Imani A, Barzegar K, Piripireivatlou S, Masomi SH. 2011. Storage of apple pollen and in vitro germination. *African Journal of Agricultural Research* **6**(3): 624-629.

Impallari FM, Monte M, Girgenti V, Del Signore MB, Sottile F 2008. Biodiversity of Sicilian Fruit Trees: Studies on Plums. *9th International Symposium on Plum and Prune Genetics, Breeding and Pomology*. Palermo, ITALY. 37-43.

Iovane M, Izzo LG, Romano LE, Aronne G. 2023. Simulated microgravity affects directional growth of pollen tubes in candidate space crops. *Frontiers in plant science* **14**.

Jain A, Shivanna KR. 1989. Loss of viability during storage is associated with changes in membrane phospholipid. *Phytochemistry* **28**(4): 999-1002.

Jain A, Shivanna KR. 1990. Storage of pollen grains of *Crotalaria retusa* in oils. *Sexual Plant Reproduction* **3**(4): 225-227.

Jalca Zambrano I, García Cruzatty LC, Castro Olaya J, Villamar Torres R, Guachambala Cando M. 2019. Condiciones óptimas para almacenamiento del polen de *Ochroma pyramidale*. *Bosque* **40**(2): 227-233.

James E, Knox R. 1993. Reproductive-Biology of the Australian Species of the Genus *Pandorea* (Bignoniaceae). *Australian Journal of Botany* **41**(5).

Jasmine SA, Richard PSS. 2020. Pollen morphology of few grasses from tropical dry evergreen forests around Chennai, Tamil Nadu. *The International Journal of Plant Reproductive Biology* **2**: 138-148.

Jeong N-R, Park K-Y. 2022. Rose Pollen Management Methods to Improve Productivity. *Agronomy* **12**(6).

Jia W-q, Wang S-p, Fan Z-q, Li J-y. 2015. Storage Condition and Viability Change of *Camellia japonica* Pollen. *Forest Research* **28**(3): 374-379.

Jia W, Wang Y, Mi Z, Wang Z, He S, Kong D. 2022. Optimization of culture medium for in vitro germination and storage conditions of *Exochorda racemosa* pollen. *Frontiers in plant science* **13**.

Jiang J, Li X, Sheng N. 1999. On the hybridization techniques of magnoliaceous plants. *Forest Research* **12**(2): 214-217.

Kadri K, Elsafy M, Makhlouf S, Awad MA. 2022. Effect of pollination time, the hour of daytime, pollen storage temperature and duration on pollen viability, germinability, and fruit set of date palm (*Phoenix dactylifera* L.) cv "Deglet Nour". *Saudi Journal of Biological Sciences* **29**(2): 1085-1091.

Kaewsri W, Paisooksantivatana Y. 2007. Morphology and palynology of *Amomum* Roxb. in Thailand. *Gard Bull Singapore* **59**: 105-112.

Kameneva LA, Koksheeva IM. 2013. Reproductive biology of seven taxa of *Magnolia* L. in the south of Russian Far East. *Bangladesh Journal of Plant Taxonomy* **20**(2): 163-170.

Kanazawa T, Kobayashi S, Yakuwa T. 1992. Flowering process, germination and storage of pollen in *Allium victorialis* L. ssp. *platyphyllum* Hult. *Journal of the Japanese Society for Horticultural Science* **60**(4): 947-953.

Kang H-G, Bae T-W, Jeong O-C, Sun H-J, Lim P-O, Lee H-Y. 2009. Evaluation of Viability, Shedding Pattern, and Longevity of Pollen from Genetically Modified (GM) Herbicide-tolerant and Wild-type Zoysiagrass (*Zoysia japonica* Steud.). *Journal of Plant Biology* **52**(6): 630-634.

Kang H-G, Chung O-C, Bae T-W, Sun H-J, Song I-J, Park KW, Lim P-O, Lee J, Lee Y-E, Song P-S, et al. 2021. Pollen-mediated flow of bar gene in transgenic herbicide-resistant turf grass *Zoysia japonica*. *Plant Biotechnology Reports* **15**(2): 241-250.

Kant R. 2019. Development of pollinium and associated changes in anther of *Calanthe tricarinata* Lindl., an epidendroid orchid. *Taiwania* **64**(3).

Karabiyik S. 2021. Effects of temperature on pollen viability and in vivo pollen tube growth in *Citrus sinensis*. *Journal of Applied Botany and Food Quality* **95**: 100-104.

Karim K, Awad MA, Manar A, Monia J, Karim A, Mohammed E. 2022. Effect of flowering stage and storage conditions on pollen quality of six male date palm genotypes. *Saudi Journal of Biological Sciences* **29**(4): 2564-2572.

Karipidis C, Olympios C, Passam HC, Savvas D. 2007. Effect of moisture content of tomato pollen stored cryogenically on in vitro germination, fecundity and respiration during pollen tube growth. *Journal of Horticultural Science & Biotechnology* **82**(1): 29-34.

Karun A, Sajini K, Niral V, Amarnath C, Remya P, Rajesh M, Samsudeen K, Jerard B, Engelmann F. 2014. Coconut (*Cocos nucifera* L.) Pollen cryopreservation. *Cryoletters* **35**(5): 407-417.

Kato K, Yamaguchi S, Chigira O, Osaka N. 2012. Flowering phenology and germination ability of pollens for *Acacia mangium* and *A. auriculiformis*. *Silvae Genetica* **61**(6): 228-236.

Khan MN, Heyne EG, Goss JA. 1971. Effect of Relative Humidity on Viability and Longevity of Wheat Pollen. *Crop science* **11**(1): 125-127.

Khan S, Perveen A. 2006. Germination capacity of stored pollen of *Abelmoschus esculentus* L. (Malvaceae) and their maintenance. *Pakistan Journal of Botany* **38**(2): 233-236.

Khan SA, Perveen A. 2006. Germination capacity of stored pollen of *Solanum melongena* L., (Solanaceae) and their maintenance. *Pakistan Journal of Botany* **38**(4): 917-920.

Khan SA, Perveen A. 2006. Germination capacity, viability and maintenence of stored pollen of *Vigna mungo* L. *International Journal of Biology and Biotechnology* **3**(4): 779-781.

Khan SA, Perveen A. 2009a. In-vitro pollen germination capacity and maintenance of *Cucumis melo* var. *melo* L. (Cucurbitaceae). *International Journal of Biology and Biotechnology* **6**(4): 229-231.

Khan SA, Perveen A. 2009b. Pollen germination capacity of three mango cultivars (*Mangifera indica* L., Anacardiaceae) from Pakistan. *Pakistan Journal of Botany* **41**(3): 1009-1012.

Khan SA, Perveen A. 2010. In vitro pollen germination capacity of *Citrullus lanatus* L., (Cucurbitaceae). *Pakistan Journal of Botany* **42**(2): 681-684.

Khan SA, Perveen A. 2011. Pollen germination capacity and viability in *Lagenaria siceraria* (Molina) standley (Cucurbitaceae). *Pakistan Journal of Botany* **43**(2): 827-830.

Khan SA, Perveen A. 2014. In vitro pollen germination of five citrus species. *Pakistan Journal of Botany* **46**(3): 951-956.

Khatun S, Flowers T. 1995. The estimation of pollen viability in rice. *Journal of Experimental Botany* **46**(282): 151-154.

Khoshkhui M, Bassiri A, Niknejad M. 1976. Effects of temperature and humidity on pollen viability of six Rose species. *Canadian Journal of Plant Science* **56**(3): 517-523.

Kim DY, Yoon MK, Do G-R, 김태일. 2009. Effects of Pollen Viability and Pistil Receptivity on Seed Set for Artificial Pollination in Strawberry. *Korean Journal of Breeding Science* **41**(4): 496-501.

Koen J, Slabbert MM, Booyse M, Bester C. 2020. Honeybush (*Cyclopia spp*.) pollen viability and surface morphology. *South African Journal of Botany* **128**: 167-173.

Koga Y, Akihama T, Fujimaki H, Yokoo M. 1971. Studies on the Longevity of Pollen Grains of Rice, *Oriza sativa* L I. Morphological change of pollen grains after shedding. *Cytologia* **36**(1): 104-110.

Korkut SS, Kazaz S, Kilic T. 2022. Different storage temperatures and times on pollen quality in cut rose varieties. *Ornamental Horticulture* **28**(2): 202-211.

Kormutak A, Bolecek P, Galgoci M, Gomory D. 2021. Longevity and germination of *Juniperus communis* L. pollen after storage. *Scientific Reports* **11**(1).

Kormuták A, Galgóci M, Bolecek P, Gomory D. 2019. Effect of storage on pollen viability in *Pinus sylvestris* L., *Pinus mugo* Turra and their hybrid swarms. *Dendrobiology* **82**: 43-51.

Kudo N, Niimi Y. 1999. Production of interspecific hybrids between *Hydrangea macrophylla* f. hortensia (Lam.) Rehd. and H-arborescens L. *Journal of the Japanese Society for Horticultural Science* **68**(2): 428-439.

Kumar KS, Mathur RK, Sparjanbabu DS. 2015. Efficacy of organic solvents for medium term storage of oil palm (*Elaeis guineensis* Jacq.) pollen. *Indian Journal of Agricultural Research* **49**(6): 516-521.

Kumar PBAN, Chaudhury R, Shivanna K. 1988. Effect of storage on pollen germination and pollen tube growth. *Current Science* **57**(10): 557-559.

Kumari M, Prasad A, Rahman LU, Mathur AK, Mathur A. 2022. In vitro germination, storage and microscopic studies of pollen grains of four *Ocimum* species. *Industrial Crops and Products* **177**.

Kundu M, Dubey A, Srivastav M, Malik S, Singh B. 2014. Effect of gamma ray irradiation and cryopreservation on pollen stainability, in vitro germination, and fruit set in Citrus. *Turkish Journal of Biology* **38**(1): 1-9.

Käpylä M. 1991. Testing the age and viability of airborne pollen. *Grana* **30**(2): 430-433.

Lanteri S, Belletti P, Lotito S. 1993. Storage of Pollen of Norway Spruce and Different Pine Species. *Silvae Genetica* **42**(2-3): 104-109.

Lee CW, Thomas JC, Buchmann SL. 1985. Factors Affecting in Vitro Germination and Storage of Jojoba Pollen. *Journal of the American Society for Horticultural Science* **110**(5): 671-676.

Lee S-Y, Oh J-Y, Joung KH, An JU, Park B-G, Kim H-D, An CG. 2020. Effect of Storage temperature on Pollen Germination and Viability of Bitter Gourd(*Momordica charantia* L.). *Journal of Agriculture & Life Science* **54**(2): 9-13.

Lei Y, Wang L, Huang F, Duan J, Luo Y, Kang Y, Yang H, Li S. 2020. Studies on Pollen Storage and vitality difference of Tea Plant varieties. *Pakistan Journal of Botany* **52**(1): 305-309.

Li B, Wang H, Liu Y 2009. Pollen Cryopreservation of *Camellia*. *1st International Symposium on Cryopreservation in Horticultural Species*. Leuven, BELGIUM. 265-268.

Li J, Shi H, Dai H, Wang Y, Zhao J, Chi Dinh N, Huang X, Sun Q. 2022. Pollen germination and hand pollination in pitaya (*Selenicereus* spp.). *Emirates Journal of Food and Agriculture* **34**(5): 369-387.

Li J, Wang Q-F, Gituru RW, Yang C-F, Guo Y-H. 2012. Reversible anther opening enhances male fitness in a dichogamous aquatic plant *Butomus umbellatus* L., the flowering rush. *Aquatic Botany* **99**: 27-33.

Li M, Jiang F, Huang L, Wang H, Song W, Zhang X, Zhang Y, Niu L. 2023. Optimization of In Vitro Germination, Viability Tests and Storage of *Paeonia ostii* Pollen. *Plants* **12**(13).

Li T-q, Liu X-f, Li Z-h, Wan Y-m, Liu X-x, Zhang X, An J, Ma H. 2018. Pollen Morphology and Characteristics of *Rhododendron longipedicellatum* (Ericaceae), an Endangered Species Endemic to Southeastern Yunnan, China. *Forest Research* **31**(3): 51-59.

Liang Yuan-Hui YC-H. 1985. Pollen Morphology of Styracaceae and Its Taxonomic Significance. *Journal of Systematics and Evolution* **23**(2): 81-90.

Lindgren K, Lindgren D. 1996. Germinability of Norway spruce and Scots pine pollen exposed to open air. *Silva Fennica* **30**(1).

Liu Q, Yang J, Wang X, Zhao Y. 2023. Studies on Pollen Morphology, Pollen Vitality and Preservation Methods of *Gleditsia sinensis* Lam. (Fabaceae). *Forests* **14**(2).

Liu X, Xiao Y, Wang Y, Chen F, Huang R, Jiang Y. 2020. The in vitro germination and storage characteristics of *Keteleeria fortunei* var. *cyclolepis* pollen provide a reference for cross breeding. *Protoplasma* **257**(4): 1221-1230.

Lora J, de Oteyza MAP, Fuentetaja P, Hormaza JI. 2006. Low temperature storage and in vitro germination of cherimoya (*Annona cherimola* Mill.) pollen. *Scientia horticulturae* **108**(1): 91-94.

Lu L, Ze-Zhi Y, Xue-Yan L, Zai-Kang T, Ya-Mei S. 2014. Pollen Germination and Storage of *Magnolia sinostellata*. *Bulletin of Botanical Research* **34**(2): 182-187.

Luza JG, Polito VS. 1985. In vitro germination and storage of English walnut pollen. *Scientia horticulturae* **27**(3-4): 303-316.

Luza JG, Polito VS. 1987. Effects of desiccation and controlled rehydration on germination *in vitro* of pollen of walnut (*Juglans spp.*). *Plant, Cell & Environment* **10**(6): 487-492.

Luza JG, Polito VS. 1988. Cryopreservation of English walnut (*Juglans regia* L.) pollen. *Euphytica* **37**(2): 141-148.

Lyakh VA, Soroka AI, Kalinova MG. 1998. Pollen storage at low temperature as a procedure for the improvement of cold tolerance in spring rape, *Brassica napus* L. *Plant Breeding* **117**(4): 389-391.

Ma Z, Bramley GLC, Zhang D, Ma Z, Bramley GLC, Zhang D. 2015. Pollen morphology of Callicarpa L. (Lamiaceae) from China and its systematic implications. *Plant Systematics and Evolution* **302**(1).

Macfarlane Smith WH, Jones JK, Sebastiampillai AR. 1989. Pollen storage of *Fragaria* and *Potentilla*. *Euphytica* **41**(1-2): 65-69.

Machado CdA, Feitosa Moura CR, Pinto de Lemos EE, Ramalho Ramos SR, Ribeiro FE, Ledo AdS. 2014. Pollen grain viability of coconut accessions at low temperatures. *Acta Scientiarum* **36**(2): 227-232.

Mack CL, Milne LA. 2016. New Banksieaeidites species and pollen morphology in Banksia. *Australian Systematic Botany* **29**(5).

Macovei A, Caser M, Dona M, Valassi A, Giovannini A, Carbonera D, Scariot V, Balestrazzi A. 2016. Prolonged Cold Storage Affects Pollen Viability and Germination along with Hydrogen Peroxide and Nitric Oxide Content in *Rosa hybrida*. *Notulae Botanicae Horti Agrobotanici Cluj-Napoca* **44**(1): 6-10.

Maeda T, Kumagai M, Inoue N, Uragami A, Ito K 2001. Effective method for seed production of all-male asparagus hybrids in a greenhouse. *10th International Asparagus Symposium*. Niigata Univ, Niigata, Japan. 133-138.

Maeda T, Yonemoto Y, Higuchi H, Hagiwara S, Taniguchi M, Humuro M, Shimizu K. 2008. Pollen Preservation Method for Japanese Pepper (*Zanthoxylum piperitum* (L.) DC.). *Horticultural Research* **7**(4): 537-541.

Maguire TL, Sedgley M. 1997. Storage temperature affects viability of *Banksia menziesii* pollen. *Hortscience* **32**(5): 916-917.

Maledon E, Nudol E, Perrot C, Gravillon M-c, Rivallan R, Cornet D, Chair H, Dossa K. 2023. First report of a successful development of yam hybrids (*Dioscorea alata* L.) from lyophilized and long-term stored pollens. *bioRxiv*.

Marcellan ON, Camadro EL. 1996. The viability of asparagus pollen after storage at low temperatures. *Scientia horticulturae* **67**(1-2): 101-104.

Marchant R, Power JB, Davey MR, Chartier-Hollis JM, Lynch PT. 1992. Cryopreservation of pollen from two rose cultivars. *Euphytica* **66**(3): 235-241.

Martinez-Gomez P, Gradziel TM, Ortega E, Dicenta F. 2000. Short-term storage of almond pollen. *Hortscience* **35**(6): 1151-1152.

Martinez-Gomez P, Gradziel TM, Ortega E, Dicenta F. 2002. Low temperature storage of almond pollen. *Hortscience* **37**(4): 691-692.

Maryam, Jaskani MJ, Fatima B, Haider MS, Naqvi SA, Nafees M, Ahmad R, Khan IA. 2015. Evaluation of pollen viability in date palm cultivars under different storage temperatures. *Pakistan Journal of Botany* **47**(1): 377-381.

Mayer E, Gottsberger G. 2000. Pollen viability in the genus *Silene* (Caryophyllaceae) and its evaluation by means of different test procedures. *Flora* **195**(4): 349-353.

Members A 2007. The Australasian Pollen and Spore Atlas: Australian National University, Canberra.

Meo AA, Khan MA. 2006. Pollen morphology as an aid to the identification of Chrysanthemum species (Compositae-Anthemideae) from Pakistan. *Pakistan Journal of Botany* **38**(1): 29.

Mercy ST, Kakar SN, Verghese TM. 1979. Studies on pollen tube growth and in vitro storage of pollen grains in *Cicer arietinum* L. and *C. soongaricum* J & S. *Agricultural Research Journal of Kerala* **17**(1): 27-32.

Mesnoua M, Roumani M, Salem A. 2018. The effect of pollen storage temperatures on pollen viability, fruit set and IQ fruit quality of six date palm cultivars. *Scientia horticulturae* **236**: 279-283.

Miler N, Wozny A. 2021. Effect of Pollen Genotype, Temperature and Period of Storage on In Vitro Germinability and In Vivo Seed Set in Chrysanthemum - Preliminary Study. *Agronomy-Basel* **11**(12).

Miranda I, Clement C. 1990. Germinación y almacenamiento del polen de pejibaye (*Bactris gasipaes* H.B.K., Palmae). *REVISTA DE BIOLOGIA TROPICAL* **38**(1): 29-33.

Mishra R, Shivanna KR. 1982. Efficacy of organic solvents for storing pollen grains of some leguminous taxa. *Euphytica* **31**(3): 991-995.

Misra RS, Bajpai PN. 1975. Studies on Floral Biology of *Janum* (Java Plum) [*Syzygium cuminii* (L) Skeels]. *Indian Journal of Horticulture* **1-2**(1-2): 15-24.

Mortazavi SMH, Arzani K, Moieni A. 2010. Optimizing Storage and In vitro Germination of Date Palm (*Phoenix dactylifera*) Pollen. *Journal of Agricultural Science and Technology* **12**(2): 181-189.

Moura CRF, Machado CDA, Lédo ADS. 2015. In vitro germination and viability of pollen grain of coconut accessions. *Revista Ciencia Agronomica* **46**(2).

Mukherjee A, George J, Pillai R, Chakrabarti SK, Naskar SK, Patro R, Nayak S, Lebot V. 2016. Development of taro (*Colocasia esculenta* (L.) Schott) hybrids overcoming its asynchrony in flowering using cryostored pollen. *Euphytica* **212**(1): 29-36.

Muniraja M, Vijayalakshmi G, Naik ML, Terry R, Khan PSSV. 2020. Ultrastructural observations of anthers, staminodes, and pollen grains of mango (*Mangifera indica* L. var. *beneshan*; Anacardiaceae). *Palynology* **44**(4).

Nadarajan J, van der Walt K, Lehnebach CA, Saeiahagh H, Pathirana R. 2021. Integrated ex situ conservation strategies for endangered New Zealand Myrtaceae species. *New Zealand Journal of Botany* **59**(1): 72-89.

Nakamura J, Morita Y, Kuroda T. 2023. *Revision of Nakamura's (1980) diagnostic characters of pollen grains of Japan*. https://www.omnh.jp/collection/Pollen/Sub2_PollenMophology_English.html [accessed 10-06-2025].

Nath J, Anderson JO. 1975. Effect of freezing and freeze-drying on the viability and storage of *Lilium longiflorum* L. and *Zea mays* L. pollen. *Cryobiology* **12**(1): 81-88.

Nazish M, Althobaiti AT, Nazish M, Althobaiti AT. 2022. Palyno-Morphological Characteristics as a Systematic Approach in the Identification of Halophytic Poaceae Species from a Saline Environment. *Plants* **11**(19).

Nepi M, Pacini E. 1993. Pollination, Pollen Viability and Pistil Receptivity in *Cucurbita pepo*. *Annals of botany* **72**(6).

Niimi Y, Shiokawa Y. 1992. A study on the storage of Lilium Pollen. *Journal of the Japanese Society for Horticultural Science* **61**(2): 399-403.

Nomura Y, Kazuma T, Makara K, Nagai T. 2002. Interspecific hybridization of autumn-flowering Allium species with ornamental Alliums and the characteristics of the hybrid plants. *Scientia horticulturae* **95**(3): 223-237.

Nomura Y, Maeda M, Tsuchiya T, Makara K. 1994. Efficient Production of Interspecific Hybrids between *Allium chinense* and Edible *Allium spp.* through Ovary Culture and Pollen Storage. *Japanese Journal of Breeding* **44**(2): 151-155.

Novara C, Ascari L, La Morgia V, Reale L, Genre A, Siniscalco C. 2017. Viability and germinability in long term storage of *Corylus avellana* pollen. *Scientia horticulturae* **214**: 295-303.

Nowicke JW, Skvarla JJ. 1984. Pollen morphology and the relationships of *Simmondsia chinensis* to the order Euphorbiales. *American Journal of Botany* **71**(2): 210-215.

Osborne R, Robbertse PJ, Claassen MI. 1992. The longevity of cycad pollen in storage. *South African Journal of Botany* **58**(4): 250-254.

Owuor BO, Owino F. 1993. Control pollination and pollen management in *Sesbania sesban* (L.) Merr. *Euphytica* **70**(3): 161-165.

Ozaki Y, Tashiro T, Kurahashi T, Okubo H. 1999. Variation of pollen viability and storability in asparagus (*Asparagus officinalis* L.) cultivars. *Journal of the Faculty of Agriculture Kyushu University* **44**(1-2): 1-8.

Ozcan A. 2020. Effect of Low-temperature Storage on Sweet Cherry (*Prunus avium* L.) Pollen Quality. *Hortscience* **55**(2): 258-260.

Ozcan A, Sutyemez M, Bukucu S, Ergun M. 2019. Pollen viability and germinability of walnut: A comparison between storage at cold and room temperatures. *Fresenius Environmental Bulletin* **28**(1): 111-115.

Pacini E, Franchi G, Lisci M, Nepi M. 1997. Pollen Viability Related to Type of Pollination in Six Angiosperm Species. *Annals of botany* **80**(1): 83-87.

Palupi ER, Sidabutar RM, Wanafiah K 2015. Pollen drying and storage for hybrid seed production of cucumber (*Cucumis sativus* L.). *5th International Symposium on Cucurbits*. Cartagena, SPAIN. 163-168.

Pan CC, Liu LD, Zhao HL, Zhao XY, Zhang L, Wang LJ, Jia XJ. 2012. Pollen Biology of *Robinia pseudoacacia* on Zhen Mountain in Yantai. *Advanced Materials Research* **356-360**.

Panella L, Wheeler L, McClintock M. 2009. Long-term survival of cryopreserved sugarbeet pollen. *Journal of Sugar Beet Research* **46**(1/2): 1.

Pauldasan A, Vipin P, Durai A, Mayavel A, Gideon VA, Nicodemus A. 2023. Floral biology, pollen viability and stigma receptivity in three species of *Casuarina*. *South African Journal of Botany* **152**: 182-191.

Perry JL, Moore JN. 1985. Pollen Longevity of Blackberry Cultivars. *Hortscience* **20**(4): 737-738.

Perveen A, Ali S. 2010. Maintenance of pollen germination capacity of *Vitis vinifera* L. (Vitaceae). *Pakistan Journal of Botany* **42**(5): 3001-3004.

Perveen A, Ali S. 2019. Germination capacity and viability of stored pollen in two ornamentals species of the genus *Caesalpinia* L. (*Caesalpini oideae* - Fabaceae) and their maintenance. *Pakistan Journal of Botany* **51**(3): 1081-1083.

Perveen A, Khan SA. 2008. Maintenance of pollen germination capacity of *Malus pumila* L., (Rosaceae). *Pakistan Journal of Botany* **40**(3): 963-966.

Perveen A, Khan SA. 2009. Maintenance of pollen germination capacity of *Glycine max* (L.) Merr., (Papilionaceae). *Pakistan Journal of Botany* **41**(5): 2083-2086.

Perveen A, Khan SA, Abid R. 2007. Maintenance of pollen germination capacity of *Carica papaya* L., (Caricaceae). *Pakistan Journal of Botany* **39**(5): 1403-1406.

Petruccelli R, Giordano C, Salvatici MC, Beghe D, Rodolfi M, Fabbri A, Benelli C. 2021. Characterization and conservation of "Olivo della Strega": an ancient olive tree, precious resource for natural and cultural heritage. *Rendiconti Lincei-Scienze Fisiche E Naturali* **32**(2): 311-324.

Pfahler PL, Linskens HF. 1973. In vitro germination and pollen tube growth of maize (*Zea mays* L.) pollen VIII. Storage Temperature and Pollen Source Effects. *Planta* **111**(3): 253-259.

Pierre J, Renard M. 2002. Pollen longevity of oil seed rape. *OCL - Oilseeds & fats Crops and Lipids* **9**(1): 11-13.

Ping-Liang C, Shan-an H, Wei J. 1990. Cryopreservation of Pollen from *Eucommia ulmoides* Oliv. and *Sinojackia xylocarpa*. *Acta Botanica Sinica* **32**(4): 288-291.

Pinney K, Polito VS 1989. Olive pollen storage and in vitro germination. *1st International Symp on Olive Growing*. Cordoba, Spain. 207-210.

Pio R, Pasqual M, Chagas EA, Barbosa W, Chagas PC, Tizato LHG, Neto JEB, Neves AA, Carvalho AS, Scarpare Filho JA 2009. Cryopreservation of Peach and Nectarine Pollen Grains. *7th International Peach Symposium*. Lleida, SPAIN. 269-275.

Pogorzelec M, Serafin A, Banach-Albinska B, Szczurowska A, Parzymies M, Bronowicka-Mielniczuk U. 2016. Pollen viability of *Salix myrtilloides* L. - an endangered species in Poland. *Acta Agrobotanica* **69**(4): 1679-Article No.: 1679.

Polito VS, Luza JG. 1988a. Longevity of Pistachio Pollen Determined by in Vitro Germination. *Journal of the American Society for Horticultural Science* **113**(2): 214-217.

Polito VS, Luza JG. 1988b. Low temperature storage of pistachio pollen. *Euphytica* **39**(3): 265-269.

Prasad PVV, Boote KJ, Allen LH, Jr. 2011. Longevity and temperature response of pollen as affected by elevated growth temperature and carbon dioxide in peanut and grain sorghum. *Environmental and Experimental Botany* **70**(1): 51-57.

Pringle GJ, Murray BG. 1991. Reproductive biology of the tamarillo, *Cyphomandra betacea* (Cav.) Sendt. (Solanaceae), and some wild relatives. *New Zealand Journal of Crop and Horticultural Science* **19**(3): 263-273.

Qian X, Liu F, Niu X, Wang C, Tian M. 2014. in vitro Pollen Germination and Storage Conditions of *Calanthe tsoongiana*. *Acta Botanica Boreali-Occidentalia Sinica* **34**(2): 341-348.

Quan QM, Li Y-X. 2012. A method for mid-term storage of *Epimedium pubescens* (Berberidaceae) Pollen. *Pakistan Journal of Botany* **44**(2): 765-768.

Quinet M, Jacquemart A-L. 2020. Troubles in pear pollination: Effects of collection and storage method on pollen viability and fruit production. *Acta Oecologica-International Journal of Ecology* **105**.

Radaeski JN, Bauermann SG, Evaldt ACP, de Lima GL. 2011. Grãos de pólen das formações campestres sul-brasileiras. *Revista de Iniciação Científica da ULBRA* **1**(9).

Radaeski JN, Cunha D, Bauermann S. 2017. Diporate pollen grains of Poaceae species: High pollen resolution for reconstruction of grasslands vegetation. *Open access journal of agricultural research* **2**(3): 000135.

Rajasekharan PE, Rao TM, Janakiram T, Ganeshan S. 1994. Freeze preservation of gladiolus pollen. *Euphytica* **80**(1-2): 105-109.

Rasheed AA, Perveen A, Abid R, Qaiser M. 2016. Pollen morphology of the subfamily Arecoideae Griff. (family Arecaceae) from Pakistan and Kashmir. *Pakistan Journal of Botany* **48**(3): 1051-1060.

Rather SA, Chaudhary HK, Kaila V. 2017. Pollen Preservation Potential of *Imperata cylindrica* - an Efficient Source for Doubled Haploid Production in Wheat. *Cereal Research Communications* **45**(3): 525-534.

Reddi CS, Raju NSN, Rao MVS. 2010. Pollination and seed set in tropical wetland grasses. *Nordic Journal of Botany* **28**(3): 354-365.

Ren R, Li Z, Li B, Xu J, Jiang X, Liu Y, Zhang K. 2019. Changes of pollen viability of ornamental plants after long-term preservation in a cryopreservation pollen bank. *Cryobiology* **89**: 14-20.

Ren R, Li Z, Zhang L, Zhou H, Jiang X, Liu Y. 2021. Enzymatic and nonenzymatic antioxidant systems impact the viability of cryopreserved *Paeonia suffruticosa* pollen. *Plant Cell Tissue and Organ Culture* **144**(1): 233-246.

Ren R, Li Z, Zhou H, Zhang L, Jiang X, Liu Y. 2020. Changes in apoptosis-like programmed cell death and viability during the cryopreservation of pollen from *Paeonia suffruticosa*. *Plant Cell Tissue and Organ Culture* **140**(2): 357-368.

Rhee HK, Lim JH, Cho HR, van Tuyl JM 2002. The relationship of storage and viability of lily pollen. *26th International Horticultural Congress*. Toronto, Canada. 319-324.

Ribeiro GS, Jose ARS, Hojo Rebouras TN, Fernandes Amaral CL. 2007. Aspectos da biologia floral relacionados à produção de frutos de pinha (*Annona squamosa* L.). *Acta Scientiarum Biological Sciences* **29**(4): 369-373.

Robles-González MM, Carrillo-Medrano SH, Cortez-Arroyo AR, Medina Urrutia VM. 2019. Viabilidad y longevidad del polen en genotipos de limón mexicano estimada mediante germinación in vitro. *Revista mexicana de ciencias agrícolas* **10**(4): 911-920.

Rodriguez-Garay B, Barrow JR. 1986. Short-term storage of cotton pollen. *Plant Cell Reports* **5**(5): 332-333.

Roubik DW, Jorge Enrique Moreno P, Roubik DW, Jorge Enrique Moreno P. 1992. Pollen and Spores of Barro Colorado Island. *Kew Bulletin* **47**(4).

Ruiz-Domínguez C, Vovides AP, Sosa V. 2019. Systematic relevance of pollen morphology in tribe Hylocereeae (Cactaceae). *PhytoKeys* **128**.

Sahar N, Spiegel-Roy P. 1980. Citrus Pollen Storage. *Hortscience* **15**(1): 81-82.

Said C, Villar M, Zandonella P. 1991. Ovule receptivity and pollen viability in Japanese larch (*Larix leptolepis* Gord.). *Silvae Genetica* **40**(1): 1-6.

Salles Pio LA, Ramos JD, Pasqual M, Junqueira KP, Santos FC, Morais Rufini JC. 2007. Viability of citrus pollen in different storage conditions. *Ciencia E Agrotecnologia* **31**(1): 147-153.

Sato S, Katoh N, Iwai S, Hagimori M. 1998. Establishment of reliable methods of in vitro pollen germination and pollen preservation of *Brassica rapa* (syn. *B. campestris*). *Euphytica* **103**(1): 29-33.
Schwartz-Tzachor R, Eisikowitch D, Dafni A. 2008. Flower characteristics and breeding system of two phenological ecotypes of *Cyclamen persicum* Mill. (Myrsinaceae) in Israel. *Plant Systematics and Evolution* **274**(1-2): 127-134.
Sedgley M, Harbard J. 1993. Pollen storage and breeding system in relation to controlled pollination of four species of *Acacia* (Leguminosae: Mimosoideae). *Australian Journal of Botany* **41**(5): 601-609.
Setia N, Grewal M, Malik CP. 1990. Studies on the Sponge Gourd (*Luffa cylindrica*) Pollen during Storage at Different Humidity Levels. *Phyton, Annales Rei Botanicae* **30**(1): 75-81.
Severova E, Kopylov-Guskov Y, Selezneva Y, Karaseva V, Yadav SR, Sokoloff D, Severova E, Kopylov-Guskov Y, Selezneva Y, Karaseva V, et al. 2022. Pollen Production of Selected Grass Species in Russia and India at the Levels of Anther, Flower and Inflorescence. *Plants* **11**(3): 285.
Shankar M, Gowthami R, Tripathi K, Deepak DA, Barpete S, Agrawal A. 2023. In vitro germination and cryopreservation technique for long-term pollen conservation of underutilized legume: Grasspea (*Lathyrus sativus*). *Indian Journal of Agricultural Sciences* **93**(2): 205-209.
Shashikumar S, Ganeshan S, Tejavathi DH 2005. Fertilizing ability of cryopreserved pollen in inter-intra specific crosses in *Carica papaya* L. *1st International Symposium on Papaya*. Genting Highlands, MALAYSIA. 73-+.
Shchori YG, Tal, Ben-Jaacov J. 1992. Pollen germination and Storage in Banksia and some other Proteaceae plants. *Acta Horticulturae* **316**: 19-22.
Shekari A, Nazeri V, Shokrpour M. 2016. Pollen viability and storage life in *Leonurus cardiaca* L. *Journal of Applied Research on Medicinal and Aromatic Plants* **3**(3): 101-104.
Shivanna KR, Heslop-Harrison J. 1981. Membrane State and Pollen Viability. *Annals of botany* **47**(6): 759-770.
Singh TJ, Gupta T. 2017. Studies of the Reproductive Biology of *Toona ciliata* M. Roem under the Himachal Pradesh Condition. *International Journal of Bio-resource and Stress Management* **8**(3).
Song J, Tachibana S. 2007. Loss of viability of tomato pollen during long-term dry storage is associated with reduced capacity for translating polyamine biosynthetic enzyme genes after rehydration. *Journal of Experimental Botany* **58**(15-16): 4235-4244.
Song ZP, Lu BR, Chen JK. 2001. A study of pollen viability and longevity in *Oryza rufipogon*, *O. sativa*, and their hybrids. *International Rice Research Notes* **26**(2): 31-32.
Sparks D, Yates IE. 2002. Pecan Pollen Stored Over a Decade Retains Viability. *Hortscience* **37**(1): 176-177.
Stebler T. 2025. *Caladium bicolor*. https://pollen.tstebler.ch/MediaWiki/index.php?title=Caladium_bicolor [accessed 10-06-2025].
Sudha R, Niral V, Samsudeen K, Aparna V, Selvamani V, Neema M. 2022. An insight into pollen morphology and evaluation of pollen viability, germination and mineral composition of some coconut (*Cocos nucifera* L.) genotypes. *South African Journal of Botany* **151**.
Sugiyama M, Sakata Y, Kitadani E, Morishita M, Sugiyama K 2001. Pollen storage for production of seedless watermelon (*Citrullus lanatus*) using soft-X-irradiated pollen. *2nd International Symposium on Cucurbits*. Tsukuba, Japan. 269-272.
Sukhvibul N, Considine JA. 1993. Medium and long term storage of *Anigozanthos manglesii* (D. Don) pollen. *New Zealand Journal of Crop and Horticultural Science* **21**(4): 343-347.

Sunilkumar K, Mathur RK, Babu DSS. 2017. Differential pollen longevity in Dura and Pisifera oil palm (*Elaeis guineensis*) fruit types at storage temperatures. *Indian Journal of Agricultural Sciences* **87**(7): 893-898.

Takatsu Y, Kasumi M, Manabe T, Hayashi M, Inoue E, Marubashi W, Niwa M. 2001. Temperature effects on interspecific hybridization between *Gladiolus* x *grandiflora* and *G. tristis*. *Hortscience* **36**(2): 341-343.

Tangmitcharoen S, Owens JN. 1997a. Floral Biology, Pollination, Pistil Receptivity, and Pollen Tube Growth of Teak (*Tectona grandis* Linn f.). *Annals of botany* **79**(3).

Tangmitcharoen S, Owens JN. 1997b. Pollen Viability and Pollen-tube Growth Following Controlled Pollination and their Relation to Low Fruit Production in Teak (*Tectona grandis* Linn. f.). *Annals of botany* **80**(4): 401-410.

Tonfack LB, Foamouhoue EN, Tchoutang DN, Youmbi E. 2019. Application of pesticide combinations on watermelon affects pollen viability, germination, and storage. *Journal of Applied Biology & Biotechnology* **7**(6): 35-39.

Tong LR, Song Y, Wang P, Wang J, Ni SG, Xia FS. 2021. Pollination Biology of *Lespedeza davurica*. *Legume Research* **44**(7): 834-837.

Towill LE. 1984. Seed set with potato pollen stored at low temperatures. *American Potato Journal* **61**(9): 569-575.

Tran XT, Parks SE, Nguyen MH, Roach PD. 2021. Reduced Pollination Efficiency Compromises Some Physicochemical Qualities in Gac (*Momordica cochinchinensis* Spreng.) Fruit. *Agronomy-Basel* **11**(1).

Udkhiawati I, Solichatun S, Pitoyo A. 2020. Characterization of pollen development and female reproductive structure of *Phalaenopsis amabilis* after pollination. *Cell Biology and Development* **4**(1).

Ullah I, Ahmad M, Jabeen A, Yusuf MO, Arfan M, Kilic O, Bagci E, Zafar M, Sultana S, Khan S, et al. 2021. Palyno-morphological characterization of selected allergenic taxa of family Poaceae from Islamabad-Pakistan using microscopic techniques. *Microscopy Research and Technique* **84**(11).

Vaknin Y, Eisikowitch D. 2000. Effects of short-term storage on germinability of pistachio pollen. *Plant Breeding* **119**(4): 347-350.

Vale EDM, Moreira SOG, Vasconcelos LFL, Guimarães ARC, Costa MDPSD. 2016. Conservação e desengorduramento de grãos de pólen de bacurizeiro. *Pesquisa Agropecuaria Brasileira* **51**(2): 192-195.

Van Bilsen DGJL, Hoekstra FA. 1993. Decreased Membrane Integrity in Aging *Typha latifolia* L. Pollen (Accumulation of Lysolipids and Free Fatty Acids). *Plant Physiology* **101**(2): 675-682.

Van Bilsen DGJL, Van Roekel T, Hoekstra FA. 1994. Declining viability and lipid degradation during pollen storage. *Sexual Plant Reproduction* **7**(5): 303-310.

van der Maas HM, Zaal MACM, De Jong ER, Van Went JL, Krens FA. 1993. Optimization of isolation and storage of sperm cells from pollen of perennial ryegrass (*Lolium perenne* L.). *Sexual Plant Reproduction* **6**(1): 64-70.

Van Der Walt ID, Littlejohn GM. 1996. Storage and viability testing of *Protea* pollen. *Journal of the American Society for Horticultural Science* **121**(5): 804-809.

Vargas DP, Souza SAM, Anjos E Silva SD, Bobrowski VL. 2009. Análise dos graos de pólen de diferentes cultivares de manona (*Ricinus communis* L., Euphorbiaceae): Conservacao e viabilidade. *Arquivos do Instituto Biológico* **76**(1): 115-120.

Veluru A, Prakash K, Neema M, Muralikrishna KS, Samsudeen K, Chandran KP, Rajesh MK, Karun A. 2021. Pollen storage of coconut dwarf accession Chowghat Orange Dwarf at low temperature. *Indian Journal of Agricultural Sciences* **91**(2): 141-144.

Vergano G, Radicati L, Martino I 1989. Investigations on viability and germinability of English Walnut pollen. *1st International Symp on Walnut Production*. Budapest, Hungary. 285-293.

Vieira CT, da Silva Rosado SC, Pereira FJ. 2020. Morfologia e viabilidade de grãos de pólen de *Toona ciliata* M. Roemer (Meliaceae) em diferentes estádios florais e tempos de armazenamento. *Scientia Forestalis* **48**(128).

Vieira LdJ, Soares TL, Rossi ML, Alves AAC, Santos FdARd, Souza FVD. 2012. Viability, production and morphology of pollen grains for different species in the genus *Manihot* (Euphorbiaceae). *Acta Botanica Brasilica* **26**.

Vishwakarma PK, Vincent L, Vasugi C, Rajasekharan PE. 2021. Effect of cryopreservation on pollen viability, fertility and morphology of different *Psidium* species. *Cryobiology* **98**: 112-118.

Visser T, De Vries DP, Welles GWH, Scheurink JAM. 1977. Hybrid Tea-rose pollen. I. Germination and storage. *Euphytica* **26**(3): 721-728.

Visser T, Oost EH. 1981. Pollen and pollination experiments. III. The viability of apple and pear pollen as affected by irradiation and storage. *Euphytica* **30**(1): 65-70.

Walt IDvd, Littlejohn GM. 1996. Pollen morphology, male hybrid fertility and pollen tube pathways in Protea. *South African Journal of Botany* **62**(5).

Walyaro DJ, Van Der Vossen HAM. 1977. Pollen longevity and artificial cross-pollination in *Coffea arabica* L. *Euphytica* **26**(1): 225-231.

Wang L, Bao Y, Wang H, He C, Wang P, Sheng L, Tang Z. 2017. Slow stamen movement in a perennial herb decreases male-male and male-female interference. *Aob Plants* **9**.

Wang L, Wu J, Chen J, Fu D, Zhang C, Cai C, Ou L. 2015. A simple pollen collection, dehydration, and long-term storage method for litchi (*Litchi chinensis* Sonn.). *Scientia horticulturae* **188**: 78-83.

Wang ML, Hsu CM, Chang LC, Wang CS, Su TH, Huang YJJ, Jiang LW, Jauh GY. 2004. Gene expression profiles of cold-stored and fresh pollen to investigate pollen germination and growth. *Plant and Cell Physiology* **45**(10): 1519-1528.

Wang X, Wu Y, Lombardini L. 2021. In vitro viability and germination of *Carya illinoinensis* pollen under different storage conditions. *Scientia horticulturae* **275**.

Wang Y, Huang Z, Ma W, Liu J, Tian L, Zhou Y, Shang F, Guo P, Wang Y, Huang Z, et al. 2023. Comparative Pollen Morphology of the Genus Chaenomeles Lindl. (Rosaceae): Diagnostic Features and Implications for Taxonomy. *Diversity* **15**(9): 960.

Wang Z, Yin M, Creech DL, Yu C. 2022. Microsporogenesis, Pollen Ornamentation, Viability of Stored *Taxodium distichum* var. *distichum* Pollen and Its Feasibility for Cross Breeding. *Forests* **13**(5).

Wang ZY, Ge YX, Scott M, Spangenberg G. 2004. Viability and longevity of pollen from transgenic and nontransgenic tall fescue (*Festuca arundinacea*) (Poaceae) plants. *American Journal of Botany* **91**(4): 523-530.

Watanabe K, Takahashi B. 1989. Factors Influencing Pollen Longevity, Germination, and Tube Growth in vitro of Kiwifruit, Actinidia deliciosa cv. Matua. *Journal of the Japanese Society for Horticultural Science* **57**(4): 591-596.

Weatherhead MA, Grout BWW, Henshaw GG. 1978. Advantages of storage of potato pollen in liquid nitrogen. *Potato Research* **21**(3): 331-334.

Wen-Ji L, Su-Ping G, Ting L, Shuo Z, Pei-Wen W, Zhi-Hui Z. 2017. Flowering biological characteristics and pollen preservation of Plumbago auriculata. *Plant Science Journal* **35**(6): 874-883.

Whitehead RA. 1962. Room-temperature storage of Coconut Pollen. *Nature* **196**(4850): 190-&.

Whitehead RA. 1963. The processing of coconut pollen. *Euphytica* **12**(2): 167-177.

Wizenberg SB, Dang M, Campbell LG. 2022. Methods for characterizing pollen fitness in *Cannabis sativa* L. *PloS one* **17**(7): e0270799-e0270799.
Wu J, Li Y-g, Liu X-h, He Y-h, Shi C-g, Zhu G-q. 2014. Detection Methods of Pollen Viability and Storage Characteristics of *Styrax tonkinensis*. *Forest Research* **27**(1): 17-23.
Wu Y, Gao W, Zhou Y, Guo H. 2022. Optimization of In Vitro Germination and storage of *Armeniaca sibirica* Pollen. *Scientia horticulturae* **304**.
Xi-mei D, Bao-qiang Z, Xin G, Yan W. 2014. Pollen Viability and Preservation of *Dendrobium lindleyi*. *Forest Research* **27**(5): 657-661.
Xie W, El-Tantawy AA, Li S, Wang J, Song J, Peng L, Chen S. 2022. Pollen germination, structures, and morphologic characters after anthers cryopreservation of *Rhododendron delavayi* Franch. *European Journal of Horticultural Science* **87**(2).
Xing G, Qu L, Zhang W, Zhang Y, Yuan X, Lei J. 2020. Study on interspecific hybridization between tulip cultivars and wild species native to China. *Euphytica* **216**(4).
Xiong H, Yuan D, Deng Z-Y, Niu G, Zou F. 2020. Effects of storage temperature and duration on pollen grain viability and pollen-tube elongation in Chinese chinqapin (*Castanea henryi* Skan). *Bangladesh Journal of Botany* **49**(2): 297-304.
Xiulian Y, Yanqing L, Lianggui W. 2015. Cold Storage and Physio-biochemical Characteristics of *Clerodendrum trichotomum* Thumb Pollen. *Acta Botanica Boreali-Occidentalia Sinica* **35**(9): 1808-1814.
Xu J, Li L, Liu Q, Shi Y, Peng J, Jia M, Liu Y. 2014. Wide-scale Pollen banking of ornamental plants through cryopreservation. *Cryoletters* **35**(4): 312-319.
Yabuya T. 1983. Pollen storage of Iris ensata thunb. in organic solvents and dry air under freezing. *Japanese Journal of Breeding* **33**(3): 269-274.
Yajima M, Watanabe Y, Yanagisawa K, Shomura S, Chino S, Oyamada S, Sato S, Torra-Reventos M, Yamaura I, Yamanaka S. 2003. Comparison of pollen characteristics, meiotic division and chromosome pairing between diploid and tetraploid Shinano walnut (*Juglans regia* L. cv. Mitsuru). *Journal of the Japanese Society for Horticultural Science* **72**(2): 134-140.
Yan GJ, Croxford B, Sedgley R 2001. Interspecific hybridisation of Leucadendron. *20th International EUCARPIA Symposium - Section Ornamentals*. Melle, Belgium. 55-63.
Yang R, Wang J, Gao W, Jiang Y, Su J, Sun D, He G. 2021. Research on the reproductive biological characteristics of *Amomum villosum* Lour. and *Amomum longiligulare* T. L. Wu. *PloS one* **16**(8).
Yang Z, Villar M. 1990. Influence of environmental conditions on the pollen vitalities of Poplars. *Forest Research* **3**(4): 388-392.
Yates IE, Sparks D, Connor K, Towill L. 1991. Reducing Pollen Moisture Simplifies Long-term Storage of Pecan Pollen. *Journal of the American Society for Horticultural Science* **116**(3): 430-434.
Yi WG, Law E, Wetzstein HY. 2003. Polyester and nylon powders used as pollen diluents preserve pollen germination and tube growth in controlled pollinations. *Sexual Plant Reproduction* **15**(5): 265-269.
Youmbi E, Akoa A, Eteme RA. 2004. In vitro germination and effects of drying time on the preservation of *Canarium schweinfurthii* pollen. *Journal of Tropical Forest Science* **16**(3): 357-362.
Youmbi E, Cerceau-Larrival MT, Verhille AM, Carbonnier-Jarreau MC. 1998. Morphologie et germination in vitro du pollen de *Dacryodes edulis* (Burseraceae). *Grana* **37**(2): 87-92.
Youmbi E, Tabi K, Ebongue N, Frank G, Tonfack L, Ntsomboh G. 2015. Oil Palm (*Elaeis guineensis* Jacq.) improvemnt: Pollen assessment for better conservation and germination. *Journal of Palm Oil Research* **27**(3): 212-219.

Youmbi E, The C, Tedjacno A. 2005. Conservation of the germination capacity of pollen grains in three varieties of maize (*Zea mays* L.). *Grana* **44**(3): 152-159.
Yuan S-C, Chin S-W, Lee C-Y, Chen F-C. 2018. *Phalaenopsis* pollinia storage at sub-zero temperature and its pollen viability assessment. *Botanical Studies* **59**.
Zambon CR, de Oliveira da Silva LF, Pio R, Bianchini FG, de Oliveira AF. 2018. Storage of pollen and properties of olive stigma for breeding purposes. *Revista Ciencia Agronomica* **49**(2): 291-297.
Zhang C-J, Mahoney J, Kim D-S, Sun S, Gan L, Fan J, Yan X. 2020. Pollen longevity, flowering phenology, and seedbank persistence of *Camelina sativa* (L.) Crantz and congenic species. *Industrial Crops and Products* **156**.
Zhang J-M, Lu X-X, Xin X, Yin G-K, He J-J, Huang B, Jiang D, Chen X-L. 2017. Cryopreservation of Citrus anthers in the National Crop Genebank of China. *In Vitro Cellular & Developmental Biology - Plant* **53**(4): 318-327.
Zhang MingYing ZM, Lu Lu LL, Wortley A, Wang Hong WH, Li DeZhu LD, Blackmore S. 2017. Evolution of angiosperm pollen: 4. Basal eudicots. *Annals of the Missouri Botanical Garden* **102**(1): 141-182.
Zhang T, Tan D-Y. 2008. Adaptive significances of sexual system in andromonoecious *Capparis spinosa* (Capparaceae). *Journal of Systematics and Evolution* **46**(6).
Zhang X-l, Gituru RW, Yang C-f, Guo Y-h. 2010. Exposure to water increased pollen longevity of pondweed (*Potamogeton spp*.) indicates different mechanisms ensuring pollination success of angiosperms in aquatic habitat. *Evolutionary Ecology* **24**(4): 939-953.
Zhang Y-L, Chen R-D, Huang C-J, Yan L. 2009. Cryo-banking of *Prunus mume* pollen and its application in cross-breeding. *Cryoletters* **30**(3): 165-170.
Zhang Y-l, Tian Z-k, Liu Y. 2006. Preservation methods of pollen of *Magnolia soulangeana* soul.-bod. *Journal of Tropical and Subtropical Botany* **14**(4): 318-320.
Zhang Y, Xiong Y, Liu Y, Huang X. 2021. Pollen morphological analysis of papaya (*Carica papaya* L.). *Crop Breeding and Applied Biotechnology* **21**(3).
Zhang Yu-Long WF-H, Chien Nan-Feng. 1988. A Study on Pollen Morphology of *Eucommia ulmoides* Oliver. *Journal of Systematics and Evolution* **26**(5): 367-370.
Zhao H, Chen F, Wang Y, Chen S, Fang W, Guo W 2006. Study on pollen viability, longevity and pistil receptivity of self-compatible chrysanthemum with small inflorescences. *International Symposium on Ornamentals, Now!* Seoul, SOUTH KOREA. 405-411.
Zhi-Neng L, Guo-Feng L, Chun-Li L, Man-Zhu B. 2006. Studies on the pollen vitality and storage capacity in Platanus acerifolia. *Wuhan Zhiwuxue Yanjiu* **24**(1): 54-57.
Zhou J, Jin Q, Qi Q, Zhu T, Peng H, Ye H. 2022. Delaying the decline of germination ability of pecan pollen by yeast *Pichia fermentans* 15B1. *Scientia horticulturae* **304**.
Zielinski QB. 1968. Techniques for collecting, handling germinating, and storing of pollen of the filbert (*Corylus spp*.). *Euphytica* **17**(1): 121-125.
Zini LM, Carrera CS, Lattar EC, Ferrucci MS. 2017. Pollen morphology in selected species of Caricaceae with special reference to novel palynological characters. *Botany* **96**(1).
Zlesak DC, Zuzek K, Hokanson SC 2005. Rose pollen viability over time at varying storage temperatures. *4th International Symposium on Rose Research and Cultivation*. Santa Barbara, CA. 337-343.
Zulkarnain Z, Eliyanti E, Swari EI. 2019. Pollen viability and stigma receptivity in *Swainsona formosa* (G.Don) J.Thompson (Fabaceae), an ornamental legume native to Australia. *Ornamental Horticulture-Revista Brasileira De Horticultura Ornamental* **25**(2): 158-167.

Supporting information references

Page MJ, McKenzie JE, Bossuyt PM, Boutron I, Hoffmann TC, Mulrow CD, Shamseer L, Tetzlaff JM, Akl EA, Brennan SE, et al. 2021. The PRISMA 2020 statement: an updated guideline for reporting systematic reviews. *BMJ* **372**.
